# Channel gain calibrated signal space projection using variational Bayesian estimation for optically pumped magnetometers

**DOI:** 10.1162/IMAG.a.1137

**Published:** 2026-02-18

**Authors:** Keita Suzuki, Yusuke Takeda, Nobuo Hiroe, Niall Holmes, Okito Yamashita

**Affiliations:** Neural Information Analysis Laboratories, Advanced Telecommunications Research Institute International, Kyoto, Japan; RIKEN Center for Advanced Intelligence Project, Tokyo, Japan; Sir Peter Mansfield Imaging Centre, School of Physics and Astronomy, University of Nottingham, Nottingham, United Kingdom; Cerca Magnetics Limited, Nottingham, United Kingdom

**Keywords:** optically pumped magnetometers, magnetoencephalography, OPM-MEG, calibration, interference correction, SSP

## Abstract

Recent success in developing optically pumped magnetometers (OPMs) has provided flexible and innovative ways to explore the high spatio-temporal dynamics of the human brain. However, the OPM sensors now in widespread use are highly susceptible to interference magnetic fields in experimental environments. Interference fields not only contaminate the measured neural signals but also introduce channel gain non-uniformity, making it challenging to effectively remove interference signals. Therefore, we propose Variational Bayesian Calibrated Signal Space Projection (VBCSSP), a new signal space projection (SSP) method developed to explicitly incorporate channel gain non-uniformity in its formulation. VBCSSP estimates the channel gain and amplitude of interference signals using hierarchical variational Bayesian estimation. Here, the performance of VBCSSP is comprehensively evaluated using static and dynamic channel gain simulation, a matrix coils (MC) experiment, and a human experimental dataset. The results for the simulation demonstrate that VBCSSP is more robust against channel gain non-uniformity compared to SSP, even when temporal gain fluctuation exists. The results also suggest that the optimal interference model can be selected based on estimated free energy. The properties confirmed in the simulation are also validated in real sensor measurements using the MC data. Finally, in the human experimental dataset, VBCSSP enhances the agreement of the estimated dipoles with SQUID-MEG. VBCSSP is expected to facilitate OPM studies by providing improved interference shielding with channel gain estimation.

## 1 Introduction

Magnetoencephalography (MEG) is highly valued for its ability to measure human brain dynamics with excellent spatio-temporal resolution (Baillet, 2017; Hämäläinen et al., 1993; He et al., 2018). The advantages of this technology have become more appealing with the advent of optically pumped magnetometers (OPMs) (Kominis et al., 2003). The OPM is considered a next-generation MEG device due to its compactness and non-cryogenic nature, making it a more flexible and accessible tool (Boto et al., 2018; Brookes et al., 2022).

Currently, despite the recent development of gradiometer-type OPMs (Cook et al., 2024; Nardelli et al., 2020), most commercially available OPMs are still magnetometer-type sensors. These are highly susceptible to remnant interference magnetic fields in a shielding room, where such fields are orders of magnitude larger than neural signals. Consequently, effective denoising techniques are crucial for the practical application of OPMs.

Signal space projection (SSP (Uusitalo & Ilmoniemi, 1997)) has been widely used for denoising cryogenic MEGs. It originally models an interference field based on an empty room recording and projects signals into a subspace orthogonal to the interference subspace. However, the empty room recording cannot be used for OPMs because the positions of OPM sensors are different between the empty room and human brain recordings. Recently, Tierney et al. (2022) validated SSP for OPMs by modeling the interference subspace using head-centered regular spherical harmonics. In particular, SSP based on the lowest-order spherical harmonics is widely used as homogeneous field correction (HFC), effectively suppressing the homogeneous magnetic field in 3D space (Tierney et al., 2021), even with a relatively small number of channels.

However, the shielding performance of SSP is significantly reduced when inaccurate information on the channel’s position, orientation, and gain of sensitivity is used, that is, when calibration errors exist (Tierney et al., 2022). This is because mismatches between the interference model and the actual observation prevent accurate projection, leading to insufficient interference shielding and contaminating the neural signals. In the case of OPMs, even with accurate coregistration between the head and sensors, calibration errors can arise due to several factors, including variations in the probe laser (Borna et al., 2020), low-frequency drift components in the environmental magnetic field (Boto et al., 2018; Iivanainen et al., 2019), and cross-axis projection errors (Borna et al., 2022). These issues have led to errors in static sensor characteristics such as non-uniform gain across channels, orientation error from the nominal axis of the sensor, and crosstalk between different axes of the same sensor. Furthermore, problems arising from magnetic fields, rather than from static sensor characteristics, are particularly challenging due to their temporal variability.

This calibration issue can be addressed using either hardware or software approaches. The hardware approach uses the reference magnetic fields systematically generated by dipoles (Hill et al., 2025; Pfeiffer et al., 2020) or multi-axis coils (Hill et al., 2025; Iivanainen et al., 2022; Liang et al., 2025) to estimate accurate channel information. Specifically, errors such as channel positions, directions, and gains are estimated from the discrepancies between the predicted measurements of the field generated by the reference coil and actual observations. This approach offers the possibility of intrinsic correction for calibration errors, addressing not only the non-uniformity of channel gain but also more complex issues such as errors in position, orientation, and crosstalk. However, implementing these methods requires additional equipment and measurements, which compromises the simplicity and portability of OPM systems. In addition, these cannot accommodate time-varying calibration errors during the experiment.

On the other hand, the software approach is an extension of signal space separation (SSS (Taulu et al., 2004)), which models the neural signal subspace alongside interference subspace. Specifically, error-robust denoising is achieved by extending SSS in the temporal (Taulu & Simola, 2006), spatial (Helle et al., 2021), or spatio-temporal (Sekihara et al., 2016; Tierney et al., 2024) domains. In particular, Tierney et al. (2024) demonstrated that their spatio-temporal projection yields outstanding shielding performance even in the presence of calibration errors. However, these methods require a large number of channels for accurately modeling the neural signal subspace, since OPMs are closer to the cortex than cryogenic systems—providing higher spatial resolution of neural signals—and thus necessitate an even greater channel count (Tierney et al., 2022). Tierney et al. (2024) proposed the adaptive multipole models (AMM), which model the neural signal subspace with spheroidal harmonics, and reported that AMM can greatly reduce the number of channels. Nevertheless, in the case of a single-axis system, AMM requires at least 9th-order harmonics; that is, it still requires a minimum of 99 channels to estimate 99 coefficients.

Accordingly, we propose a new SSP method named Variational Bayesian Calibrated Signal Space Projection (VBCSSP), which explicitly incorporates channel gain non-uniformity in the SSP formulation. VBCSSP can denoise the interference field even when time-varying calibration errors exist and only a few OPM sensors are available. A novel feature of the proposed method is its Bayesian foundation, where the model is optimized by variational Bayesian estimation. Here, the algorithm can automatically reject channels with unnatural gains. Using static channel gain simulation data, we demonstrate that our method robustly suppresses interferences, even in the presence of channel gain non-uniformity and under-determined cases. Moreover, the derived free energy permits model comparisons, such as selecting the optimal order of the spherical harmonic functions. Using dynamic channel gain simulation data, we demonstrate that time-varying channel gain errors can be corrected by applying VBCSSP to segmented data. The advantages of VBCSSP are also confirmed experimentally by real sensor measurements in controlled magnetic fields by matrix coils (MC). Finally, we demonstrate the usefulness of VBCSSP using a human experimental dataset. Consequently, our method provides robust interference suppression against channel gain non-uniformity without additional costs.

This paper is organized as follows. First, we formulate the SSP and then propose its extension, VBCSSP. Next, we comprehensively investigate the performance of VBCSSP using static channel gain simulation while also considering model violations. Then, we evaluate VBCSSP using dynamic channel gain simulation. We also investigate its performance through an experiment using MC data. Finally, we evaluate the usefulness of VBCSSP for a human experiment using an open dataset, focusing on current source estimation.

## 2 Theory

In this section, we first describe SSP and then extend it to propose VBCSSP.

### 2.1 Signal space projection

SSP projects MEG data onto a subspace that is orthogonal to modeled interference space. The MEG signal observed by the M-channel OPM system $\text{b}\left(t\right)\in {\mathbb{R}}^{M\times 1}$ and N-dimensional interference amplitudes $\text{h}\left(t\right)\in {\mathbb{R}}^{N\times 1}$ at time t can be described by the following model:

b(t)=Sh(t)+e(t) (1)

where $\text{S}\in {\mathbb{R}}^{M\times N}$ is a column space matrix representing an arbitrary basis set of interference subspace specified by the user and the dimension of S determines N. In the cryogenic MEG system, a low-rank basis of empty room recording is generally used for S. However, in the OPM system, the identity of the channel’s position and orientation is not maintained between the empty room and subject recordings. Therefore, the regular part of the spherical harmonics function, which approximates the magnetic field coming from outside the array, is used for OPMs (Tierney et al., 2022). For modeling the interference using the spherical harmonics basis, larger orders can represent higher spatial frequencies but require more channels. In this study, we use the spherical harmonics basis for S, where the mathematical definition follows the previous study by Tierney et al. (2022). We denote the order of the spherical harmonics function for SSP as $L_{\text{SSP}},\;$ which  determines the number of interference bases by $N=L_{\text{SSP}}^{2}+2L_{\text{SSP}}$ . Here, note that SSP with $L_{\text{SSP}}=1$ is equivalent to the HFC (Tierney et al., 2021).

On the other hand, $\text{e}\left(t\right)\in {\mathbb{R}}^{M\times 1}$ represents residuals, and this term contains neural signals x(t) and system noise ε(t), allowing it to be decomposed into e(t)=x(t)+ε(t). If the neural subspace is also included in S, the method corresponds to the SSS framework (Tierney et al., 2022). By considering time series data with T points, Eq. (1) is described in matrix form as

B=SH+e, (2)

where B=(b(1),…,b(T)), H=(h(1),…,h(T)), and e=(e(1),…,e(T))  .

The objective of SSP is to remove the interference component SH and extract the residuals e. This is achieved by projecting the interference to null space as follows:

$$\begin{array}{c} \begin{array}{c} \hat{\text{e}}=\text{B}-\text{S}\hat{\text{H}},\\ \;\;\;=\text{B}-\text{S}{\text{S}}^{†}\text{B}, \end{array}\\ \begin{array}{c} \;\;\;=\left(\text{I}-\text{S}{\text{S}}^{†}\right)\text{B},\\ ={\text{P}}_{\text{SSP}}\text{B}. \end{array} \end{array}$$ (3)

Here, $\hat{\text{H}}={\text{S}}^{†}\text{B}$ is estimated amplitude for S, ${\text{S}}^{†}={\left({\text{S}}^{⊤}\text{S}\right)}^{-1}{\text{S}}^{⊤}$ is the pseudoinverse, and ${\text{P}}_{\text{SSP}}=\text{I}-\text{S}{\text{S}}^{†}$ is the projection matrix for SSP. As Eq. (3) indicates, ${\text{P}}_{\text{SSP}}$ depends only on S, the basis of the interference subspace.

### 2.2 Channel gain calibrated signal space projection using Bayesian estimation

The SSP model in Eq. (1) assumes that the gains of all channels are fixed to the same value. In practice, however, the gains fluctuate due to problems such as variations in the probe laser, low-frequency drift, and cross-axis projection errors. Such uncertainty of the gains can be modeled by incorporating the gain matrix C as follows:

b(t)=CSh(t)+e(t), (4)

where the diagonal part of C represents the channel gain and the off-diagonal part represents the crosstalk between channels. This formalization is also suggested by previous studies (Iivanainen et al., 2022; Liang et al., 2025; Tierney et al., 2024). In this study, we consider only the channel gain and define the gain matrix as diagonal: $\text{C}=\text{diag}\left({\text{c}}_{d}\right)=\text{diag}\left(c_{1},\ldots ,c_{M}\right)$, where ${\text{c}}_{d}$ is a vector of diagonal elements and each $c_{m}$ represents the gain of each channel. Our method assumes that the magnetic field is normalized to have mean 0 and standard deviation 1 for model simplicity.

To estimate C in addition to h and e, we formulate Eq. (4) as a variant of the Bayesian factor analysis (Sekihara & Nagarajan, 2015). We named the proposed method Variational Bayesian Calibrated Signal Space Projection (VBCSSP). First, for the conjugacy with the following Eqs. (6) and (7) (Bishop, 2006), we assume that the residuals e follow an identically independent Gaussian distribution $N\left(\text{e}\left(t\right)|0,\Lambda^{-1}\right)$, where Λ=λI is a diagonal noise precision matrix with the scaling parameter λ. Then, the likelihood function, which is the probabilistic model of Eq. (4), is given by

$$\begin{array}{c} P\left(\text{B}|\text{H},{\text{c}}_{d}\right)=P\left(\text{b}\left(1\right),\ldots ,\text{b}\left(T\right)|\text{h}\left(1\right),\ldots ,\text{h}\left(T\right),{\text{c}}_{d}\right),\\ =\prod_{t=1}^{T}P\left(\text{b}\left(t\right)|\text{h}\left(t\right),{\text{c}}_{d}\right),\\ =\prod_{t=1}^{T}N\left(\text{b}\left(t\right)|\text{CSh}\left(t\right),\Lambda^{-1}\right). \end{array}$$ (5)

From the Bayesian perspective, prior distributions are introduced to H and **${\text{c}}_{d}$**. The prior distribution of H is defined as

$$\begin{array}{c} P\left(\text{H}\right)=P\left(\text{h}\left(1\right),\ldots ,\text{h}\left(T\right)\right),\\ =\prod_{t=1}^{T}P\left(\text{h}\left(t\right)\right),\\ =\prod_{t=1}^{T}N\left(\text{h}\left(t\right)|\text{0},\text{I}\right). \end{array}$$ (6)

The prior distribution of ${\text{c}}_{d}$ is defined as

$$\begin{array}{c} P\left({\text{c}}_{d}\right)=N\left({\text{c}}_{d}|\text{0},{\text{D}}^{-1}\right),\\ =N\left({\text{c}}_{d}|\text{0},\rho \text{K}\right), \end{array}$$ (7)

where ${\text{D}}^{-1}=\rho \text{K}$ is the covariance matrix of ${\text{c}}_{d}$ with the scaling parameter ρ. Although we set K=I in this paper, we can easily employ the Gaussian process prior by configuring K (e.g., a spatial smoothness prior on gain estimation can be introduced by defining K based on the structural relationships among channels). For algorithmic simplicity, we set the mean of ${\text{c}}_{d}$ to 0, although the channel gains should be positive values.

Given the likelihood function Eq. (5) and the prior distributions Eqs. (6) and (7), we compute the joint posterior distribution of H and ${\text{c}}_{d}$ as follows:

$$P\left(\text{H,}{\text{c}}_{d}|\text{B}\right)=\frac{P\left(\text{B}|\text{H},{\text{c}}_{d}\right)P\left(\text{H}\right)P\left({\text{c}}_{d}\right)}{P\left(\text{B}\right)}.$$ (8)

However, this cannot be solved analytically due to the marginal likelihood P(B). Therefore, the approximated posterior distribution is calculated using the variational Bayesian method (for details, see Supplementary Mathematical Derivations A). The resulting algorithm consists of iterative updating of the variational posterior distributions Q(H|B) and $Q\left({\text{c}}_{d}|\text{B}\right)$. The estimations $\bar{H}$ and ${\bar{c}}_{d}$ are the means of the posterior distributions, and they are derived as

$$\bar{h}(t)=\Gamma^{-1}S^{T}\bar{C}\Lambda b(t)$$ (9)

and

$${\bar{c}}_{d}=\Phi^{-1}diag(SR_{hb}\Lambda ),$$ (10)

where

$$\begin{array}{l} \;\;\;\Gamma =S^{T}\bar{C^{2}}\Lambda S+I,\\ \;\;\Phi =diag\{diag(SR_{hh}S^{T}\Lambda )\}+D,\\ R_{hh}=T\Gamma^{-1}+\sum_{t=1}^{T}\left\{\bar{h}(t){\bar{h}}^{T}(t)\right\},\\ R_{hb}=\sum_{t=1}^{T}\{\bar{h}(t)b^{T}(t)\}. \end{array}$$ (11)

Here, $\bar{C}$ and $\bar{C^{2}}$ represent the expectation for C with respect to the posterior distributions, and they are calculated by Eqs. (S 11) and (S 12); Γ and Φ are the precision matrices of posterior for h (Eq. (S3)) and ${\text{c}}_{d}$ (Eq. (S4)), respectively.

The scaling parameters λ and ρ are estimated by empirical Bayes (see Supplementary Mathematical Derivations B) as follows:

$$\begin{array}{l} {\hat{\lambda }}^{-1}=\frac{1}{TM}\{\sum_{t=1}^{T}{\text{b}}^{⊤}\left(t\right)\text{b}\left(t\right)-2\sum_{t=1}^{T}{\text{b}}^{⊤}\left(t\right)\bar{\text{C}}\text{S}\bar{\text{h}}\left(t\right)\\ \;\;\;\;\;\;\;\;\;+tr\left({\text{S}}^{⊤}\bar{{\text{C}}^{2}}\text{S}{\text{R}}_{hh}\right)\} \end{array}$$ (12)

and

$$\hat{\rho }=\frac{1}{2M}tr\left\{{\text{K}}^{-1}\left(\Phi^{-1}+\bar{{\text{c}}_{d}}\;{\bar{{\text{c}}_{d}}}^{⊤}\right)\right\}.$$ (13)

Equations (9), (10), (12), and (13) are iteratively optimized. Then the projection matrix of VBCSSP is as follows:

$${\text{P}}_{VBCSSP}=\text{I}-\bar{\text{C}}\text{S}\Gamma^{-1}\bar{\text{C}}{\text{S}}^{⊤}\Lambda .$$ (14)

Compared with ${\text{P}}_{\text{SSP}}$ , ${\text{P}}_{\text{VBCSSP}}$ is L2-regularized due to the prior distribution in Eq. (6). In the neuroscience literature, free energy is one of the popularly used statistical criteria for model selection (Friston et al., 2003; Fukushima et al., 2015; Wipf & Nagarajan, 2009). In OPM studies, Lopez et al. (2019) attempted to select the optimal model of observation noise, while Duque-Muñoz et al. (2019) demonstrated the estimation of sensor geometry. For channel calibration, Iivanainen et al. (2022) also attempted to select the optimal order of spherical harmonics using a similar criterion. Therefore, we too examined whether free energy could be used for optimal order selection (see Supplementary Mathematical Derivations C for the computation of free energy).

Since the estimated values of H and C are mutually adjustable, their absolute values are meaningless. To mitigate this issue, we introduce a correction based on the norm ratio between the SSP and VBCSSP estimates:

$$\begin{array}{c} {\text{H}}_{\text{VBCSSP}}\leftarrow \alpha {\text{H}}_{\text{VBCSSP}},\\ {\text{c}}_{d}\leftarrow {\text{c}}_{d}/\alpha , \end{array}$$ (15)

where $\alpha =\;||{\text{H}}_{\text{SSP}}|{|}_{F}/\text{H}|{|}_{\text{VBCSSP}}|{|}_{F}$ is the correction factor, ${\text{c}}_{d}$ is the estimated channel gain vector by VBCSSP, ${\text{H}}_{\text{SSP}}$ and ${\text{H}}_{\text{VBCSSP}}$ are the estimated interference amplitudes by SSP and VBCSSP, $||\;{||}_{F}$ represents the Frobenius norm, and left-facing arrows indicate replacing the left side with the right side. This operation scales ${\text{H}}_{\text{VBCSSP}}$ so that its norm matches that of ${\text{H}}_{\text{SSP}}$ while preserving the magnitude of the magnetic field CSH in Eq. (4). The effect of this correction is evaluated in Supplementary Fig. S1; further details are provided in Supplementary Note 1. All subsequent estimates incorporate this correction.

Given the spherical harmonics-based interference model, it is not usual for the channel gain to be negative. Therefore, as a heuristic extension, we introduced a process to reject channels with negative estimated gain. The modified VBCSSP algorithm is as follows:

**Algorithm 1.** Modified VBCSSP algorithm

0. Initialize all parameters 1. H-step (Eq. (9)) 2. C-step (Eq. (10)) 3. Update hyperparameters (Eqs. (12) and (13)) 4. Check negative gains: Yes → Reject channels with negative gains Go to step 0 No → Go to step 1 5. Check convergence: Yes → Finish the iteration No → Go to step 1 6. Apply correction (Eq. (15))

Convergence is judged when the relative change of free energy between successive iterations falls below a predefined threshold (e.g., 0.005%).

### 2.3 Channel gain estimation by regression

Another way to solve Eq. (4) for C is to perform a least-square regression for each channel (Iivanainen et al., 2022). The following is performed for all M channels:

$${\hat{c}}_{m}={\text{b}}_{m}^{⊤}{\left({\text{s}}_{m}{\hat{\text{H}}}_{SSP}\right)}^{†},$$ (16)

where ${\hat{c}}_{m}$ is the estimated diagonal element of C, ${\hat{\text{H}}}_{\text{SSP}}={\text{S}}^{†}\text{B}$ is the estimated amplitude by SSP, and ${\text{s}}_{m}\in {\mathbb{R}}^{1\times N}\;$ is the m-th row of S. Eq. (16) can be computed for all channels at once because the pseudo-inverse of any vector v can be calculated by ${\text{v}}^{†}\;={\text{v}}^{⊤}/\;{\text{v}}^{⊤}\text{v}$ ,

$${\hat{\text{c}}}_{d}=\frac{\text{diag}\left(\text{B}{\hat{\text{B}}}_{\text{SSP}}^{⊤}\right)}{\text{diag}\left({\hat{\text{B}}}_{\text{SSP}}{\hat{\text{B}}}_{\text{SSP}}^{⊤}\right)},$$ (17)

where ${\hat{\text{B}}}_{\text{SSP}}=\text{S}{\hat{\text{H}}}_{\text{SSP}}$ , and we can see that this estimation is the ratio of the channel-wise inner products of B and ${\hat{\text{B}}}_{\text{SSP}}$ . We call this method CSSP in this paper, and it is used for a competitor benchmark against VBCSSP.

## 3 Materials and Methods

Using simulated and real data, we evaluated the performance of VBCSSP in comparison with the SSP and CSSP introduced in the theory section. In the static channel gain simulation experiments, we comprehensively investigated the performance of SSPs with and without model misspecification in the presence of static channel gain errors. Using the dynamic channel gain simulation, we evaluated SSPs under time-varying channel gain errors. We also confirmed the properties of SSPs using MC experiment data. Finally, we applied VBSSP to a publicly available human experimental dataset. Since all analyses were performed on simulated datasets or datasets from previous studies, this study did not require additional approval by an ethics committee.

### 3.1 Static channel gain simulation

We simulated a situation in which an interference field modeled by a spherical harmonics function was observed by OPM with non-uniform and temporally static channel gain. Then, we attempted to suppress the interference using SSP with a spherical harmonics basis. Since the true environmental magnetic field (true model) is unknown in actual situations, it is not possible to guarantee the selection of the order for SSP’s spherical harmonics basis. Therefore, we independently set the order of the spherical harmonics function for the true model and the SSP. Then, their effects on SSP and its variants were comprehensively investigated. In this simulation, the spherical harmonics function for the true model and the SSPs was generated using the programs of Tierney et al. (2022). The spherical harmonics function for the true model was normalized so that the norm of each column becomes 1. We denote the order of spherical harmonics as $L_{\text{SSP}}$ for SSPs and $L_{\text{true}}$ for the true model.

An observation MEG signal ${\text{B}}_{\text{sim}}\in {\mathbb{R}}^{45\times 10000}$ , measured by a 45-ch OPM system consisting of 15 tri-axial sensors with 10,000 time-points, is generated by

$${\text{B}}_{\text{sim}}={\text{C}}_{\text{true}}{\text{S}}_{\text{true}}{\text{H}}_{\text{true}}+{\text{e}}_{\text{true}}\;,$$ (18)

where ${\text{e}}_{\text{true}}=\text{GX}$ , $\text{G}\in {\mathbb{R}}^{45\times 10003}$ is a lead field matrix for 45 channels and 10,003 vertices and $\text{X}\in {\mathbb{R}}^{10003\times 10000}$ is a simulated neural signal drawn from a Gaussian distribution with mean 0, with its standard deviation scaled so that ${\text{e}}_{\text{true}}$ has a standard deviation of 100. $\;{\text{H}}_{\text{true}}\in {\mathbb{R}}^{N\times 10000}$ is also sampled from a Gaussian with mean 0, but the standard deviation is 100 times larger than ${\text{e}}_{\text{true}}$ . This simulates a signal-to-noise ratio situation: The magnitude of the interference field is 10[pT] when the magnitude of ${\text{e}}_{\text{true}}$ is 100[fT]. ${\text{S}}_{\text{true}}\in {\mathbb{R}}^{45\times N}$ is the true interference model generated by a spherical harmonics function of the first to sixth order. $\;{\text{C}}_{\text{true}}$ is a diagonal matrix, and the m-th element $c_{\text{true},\;m}$ is sampled from $N\left(1,\sigma_{C}^{2}\right),$ where $\sigma_{c}$ ranges from 0 to 0.1. This assumes that a deviation of the channel gain’s non-uniformity $100\sigma_{C}$ ranges from 0 to 10 [%]. In this simulation, we refer to this gain non-uniformity $100\sigma_{C}$ as the gain error [%]. For the sensor arrangement, we used real data from our publicly available dataset (https://vbmeg.atr.jp/nictitaku209/). Note that the original dataset employed dual-axial OPM sensors, so we made a virtual tri-axial system by adding the third axis orthogonal to the others. Under each condition, we repeated the data generation and evaluation 50 times.

### 3.2 Dynamic channel gain simulation

**Fig. 1.**
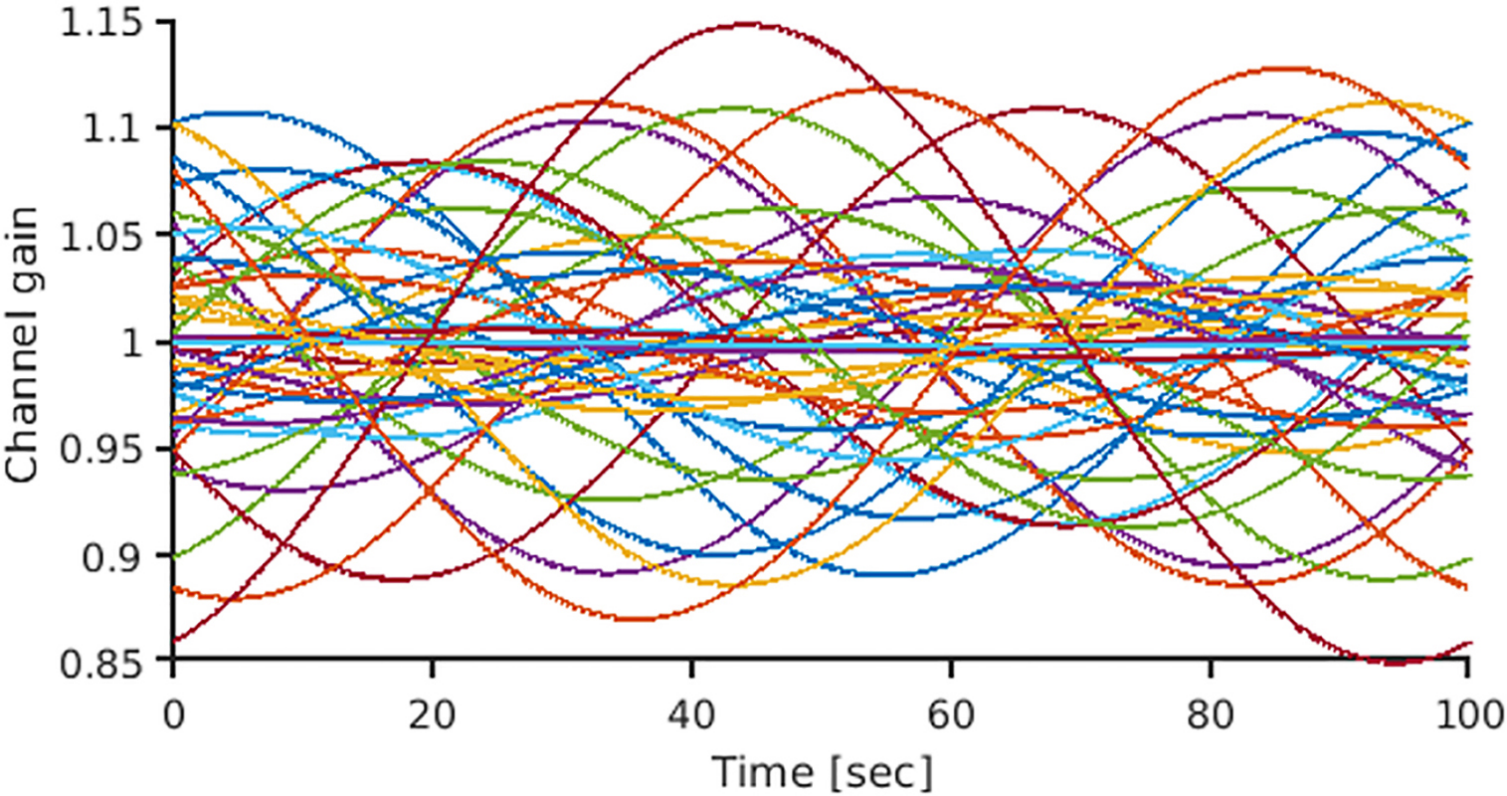
Channel gain time-series. Time series of channel gain over a 100-sec (10000 time points) simulation. Each line represents the gain time series for each channel.

Section 3.1 assumes static channel gains. However, in real environments, channel gains may also exhibit temporal fluctuations due to time-varying interferences. To explore this situation, we conducted dynamic channel gain simulation. The basic settings were identical to those detailed in Section 3.1. However, each channel gain was modeled as a sinusoidal time series, with the sine wave’s amplitude drawn from a Gaussian distribution with a mean of 1 and a standard deviation of 0.1. The frequency of the sine wave was 0.01 Hz, and each channel had a random initial phase. The simulation time was set to 100 sec, and the sampling frequency was 1000 Hz. An example of a channel gain time series is shown in Figure 1.

The observation model for dynamic gain can be written as follows:

$${\text{B}}_{\text{sim}}={\text{C}}_{\text{vary}}\circ {\text{S}}_{\text{true}}{\text{H}}_{\text{true}}+{\text{e}}_{\text{true}}\;,$$ (19)

where ${\text{C}}_{\text{vary}}=\left[{\text{c}}_{d,\text{true}}\left(1\right),{\text{c}}_{d,\text{true}}\left(2\right),\ldots ,{\text{c}}_{d,\text{true}}\left(10000\right)\right]$ is a matrix representing the time-varying channel gain, in which each column ${\text{c}}_{d,\text{true}}\left(t\right)\in {\mathbb{R}}^{45}$ represents the true gain vector at time t, and ∘ indicates the Hadamard (element-wise) product. Given this observation, the VBCSSP model of Eq. (4) is valid only at a single time point. Note that Eq. (18) corresponds to the special case in which ${\text{c}}_{d,\text{true}}\left(t\right)$ is constant across all time points t. Under each condition, we repeated the data generation and evaluation 50 times.

### 3.3 Evaluation for simulated dataset

For the static simulation, we carried out interference field suppression by SSP, CSSP, and VBCSSP. Then, we evaluated the results based on two criteria: shielding factor (SF) and correlation of residuals. Shielding factor is evaluated as follows:

$$\text{SF}=10\log_{10}\frac{\text{var}\left({\text{B}}_{\text{sim}}\right)}{\text{var}\left(\hat{\text{e}}\right)},$$ (20)

where $\text{var}\left({\text{B}}_{\text{sim}}\right)$ is the variance of simulated data and $\text{var}\left(\hat{\text{e}}\right)$ is the variance of the estimated residual by each denoising method, meaning the variance after denoising. The more the interference component is removed from the observed signal, the larger SF becomes. On the other hand, Eq. (20) can also be increased by simply decreasing the denominator, which occurs in a badly conditioned inverse matrix. Therefore, we also evaluated the correlation between the true and estimated residuals:

$$\text{CORR}\left(\text{e}\right)=\text{corr}\left({\text{e}}_{\text{true}},\hat{\text{e}}\right),$$ (21)

where corr() is computed by calculating the correlation coefficients of time series for individual channels and averaging them across all channels. Note that the residual ${\text{e}}_{\text{true}}$ is the observed neural signals in the simulation. For CSSP and VBCSSP, the correlation between the true and estimated channel gains was also evaluated:

$$\text{CORR}\left(\text{C}\right)=\text{corr}\left(\text{diag}\left({\text{C}}_{\text{true}}\right),\text{diag}\left(\hat{\text{C}}\right)\right).$$ (22)

For VBCSSP, we also evaluated free energy after convergence.

Because interference modeled by spherical harmonics can also span the neural subspace, any SSP-based projector may remove neural signals (Tierney et al., 2022, 2024). Accordingly, we assessed lead field attenuation after SSP, CSSP, and VBCSSP. For each cortical vertex i, the attenuation was computed as follows (Tierney et al., 2022):

$${\text{attenuation}}_{i}=10\log_{10}\frac{\text{var}\left(\text{P}{\text{G}}_{i}\right)}{\text{var}\left({\text{G}}_{\text{i}}\right)},$$ (23)

where ${\text{G}}_{i}$ is a column of lead field matrix and P is the projection matrix by SSP, CSSP, or VBCSSP.

For the dynamic simulation, we applied SSP, CSSP, and VBCSSP to temporally segmented data using window lengths of [5, 10, 20, 50, 100] sec. For example, for a 5-sec window, the 100-sec simulation was divided into 20 segments, whereas for a 100-sec window, each method was applied to all data. For each segment, we evaluated CORR(e) and CORR(C) (except for SSP) without model misspecification (i.e., $L_{\text{SSP}}=L_{\text{true}}$ ). The scores were averaged across segments. $L_{\text{SSP}}$ was investigated from 1 to 6, as in the static simulation.

### 3.4 MC experiment

For the empirical data benchmarking, we applied each method to data collected from controlled interference generated by MC active magnetic shielding system (Holmes, 2023) embedded in the walls of a magnetically shielded room at the University of Nottingham, UK. This experiment was originally conducted to calibrate channel positions, orientations, and gains using a known reference field (Hill et al., 2025; Iivanainen et al., 2022). For our analysis, focused solely on gain calibration, channel positions and orientations were pre-calibrated according to the calibration method proposed by Hill et al. (2025). Briefly, the MC system comprises 94 individual coils. A field mapping process derives the field produced by each coil over a desired volume, and the coil currents can be chosen so that the superposition of fields from each coil produces the desired spatial pattern. In this case, the coils were driven such that three uniform fields and five field gradients were produced simultaneously. The uniform fields are used to identify the gains of the channels, and the gradient fields use this information to infer channel position. Each spatial pattern was produced at a different frequency between 3 and 10 Hz in steps of 1 Hz. The signals were recorded by 57 triaxial OPM sensors (QuSpin Inc.) at 1,500 Hz over a 14-sec period.

We applied SSP, CSSP, and VBCSSP with $L_{\text{SSP}}=1\sim 5$ and evaluated SF . For CSSP and VBCSSP, we computed CORR(C) by treating the channel gains estimated by Hill et al. (2025) as the ground truth. In addition, we assessed the free energy estimated by VBCSSP.

### 3.5 Human experimental data

In this section, we evaluated the performance of VBCSSP using our published dataset (https://vbmeg.atr.jp/nictitaku209/) and compared it with that of SSP. From this dataset, we used the auditory and somatosensory tasks performed by four subjects and measured by 15 dual-axial OPM sensors (total 30 channels). A key feature of this dataset is that each subject has both OPM-MEG and SQUID-MEG data. Therefore, we evaluated SSP and VBCSSP for the proximity of estimated dipoles between OPM and SQUID. We also evaluated SF at the frequency where prominent interference signals appear (6–10 Hz). For this dataset, SF at frequency f is calculated by

$$\text{SF}\left(f\right)=10\log_{10}\frac{\text{psd}\left({\text{B}}_{\text{pre}},f\right)}{\text{psd}\left({\text{B}}_{\text{post}},f\right)},$$ (24)

where psd(B,f) indicates the computing power spectrum density at frequency f and ${\text{B}}_{\text{pre}}$ and ${\text{B}}_{\text{post}}$ are MEG signals before and after SSPs, respectively.

The analysis was carried out using the scripts for the dataset (https://vbmeg.atr.jp/docs/v30/static/vbmeg3_ose_tutorial.html) in the following pipeline: manual channel rejection, detrending, regress-out EOGs, prefiltering (bandpass 1–100 Hz, bandstop 57–63, and 117–123 Hz), SSP, filtering (bandpass 6–50 Hz), trial-segmentation, bad trial rejection, baseline correction, and dipole fitting. SSP in the pipeline was replaced by SSP or VBCSSP. For details on the SQUID-MEG analysis, refer to the above tutorial page.

## 4 Results

In this section, we report the results of evaluation using static and dynamic simulation, an MC experiment, and a human experimental dataset.

### 4.1 Static channel gain simulation

#### 4.1.1 Evaluating shielding factor and correlation of residuals

**Fig. 2.**
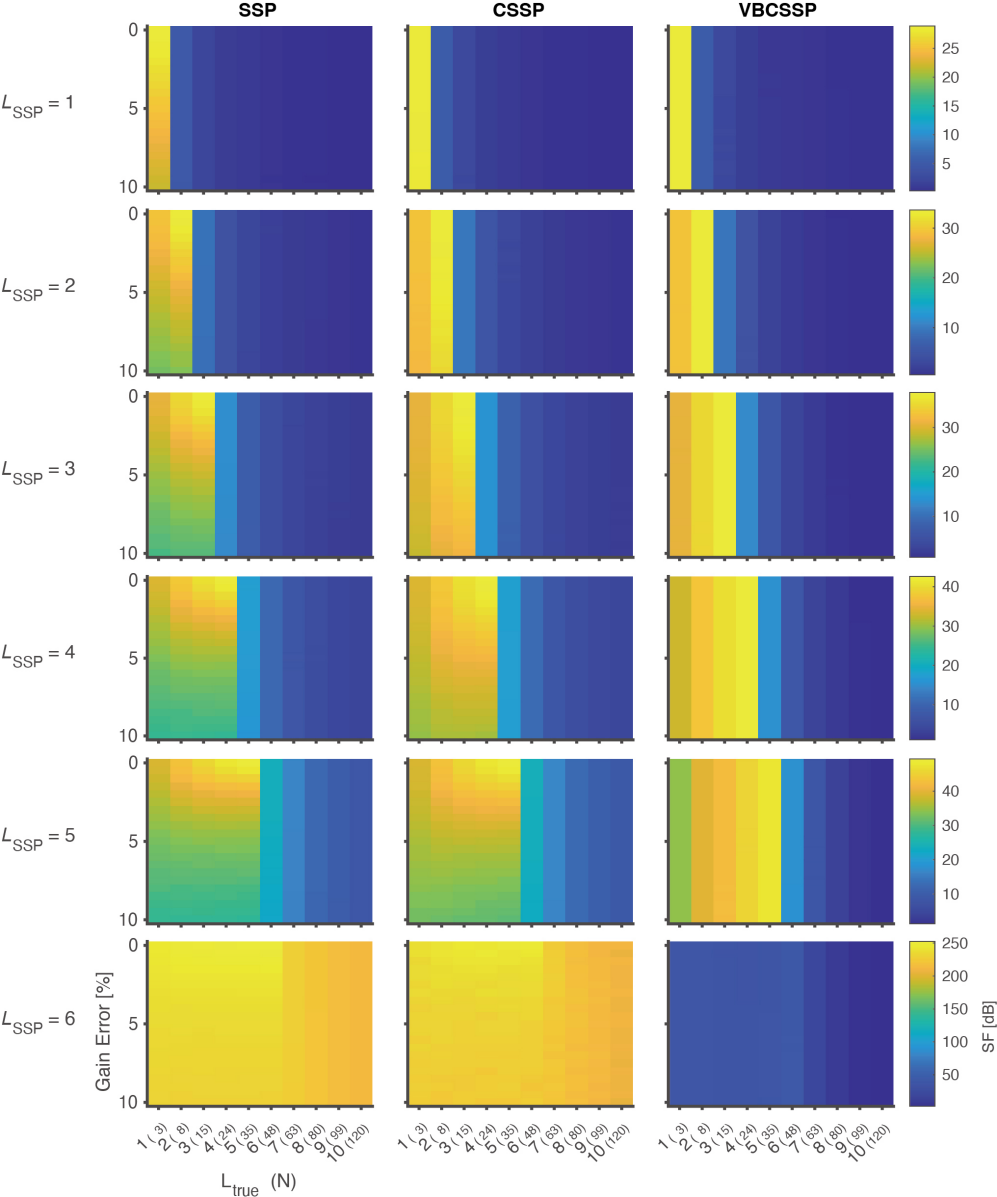
Shielding factor for static simulation. Each panel in the figure visualizes the shielding factor SF for each method with a specific order of spherical harmonics ${\text{L}}_{\text{SSP}}$ . The horizontal axis of a panel is the order of the true model ${\text{L}}_{\text{true}}$ , and the vertical axis is the magnitude of gain error $100\sigma_{\text{C}}$ [%]. The number in parentheses on the horizontal axis represents the number of bases N for the true interference model $S_{true}$ , computed by $L_{true}^{2}+2L_{true}$ . ${\text{L}}_{\text{SSP}}$ varies along a column of panels from first to sixth, while a row represents the results for SSP, CSSP, and VBCSSP. Colored bars are scaled for each row.

**Fig. 3.**
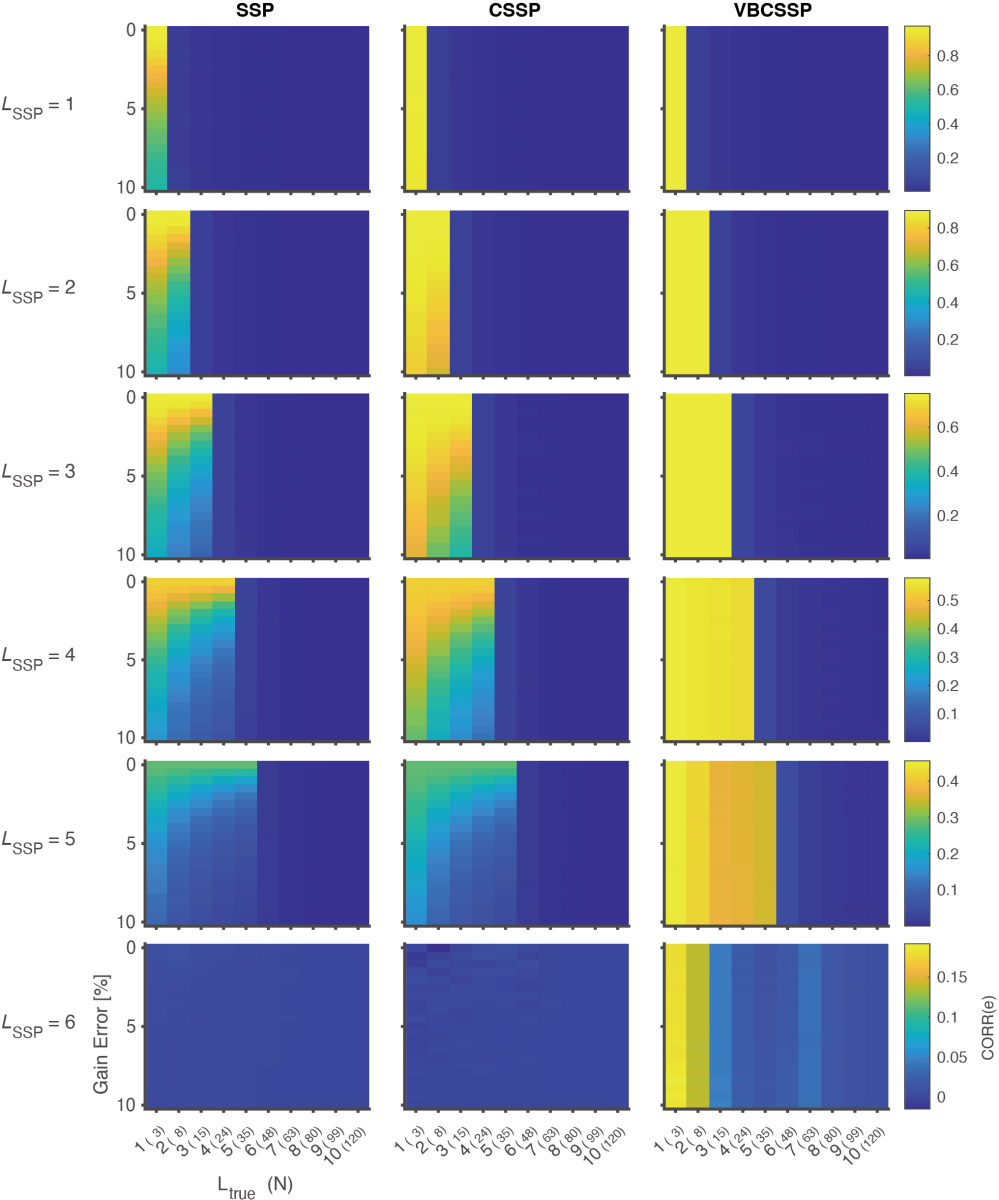
Similarity between true and estimated residuals for static simulation. Each panel in the figure visualizes the correlation of residuals CORR(e) for each method with a specific order of spherical harmonics ${\text{L}}_{\text{SSP}}$ . The horizontal axis of a panel is the order of the true model ${\text{L}}_{\text{true}}$ , and the vertical axis is the magnitude of gain error $100\sigma_{\text{C}}$ [%]. The number in parentheses on the horizontal axis represents the number of bases N for the true interference model $S_{true}$ , computed by $L_{true}^{2}+2L_{true}$ . ${\text{L}}_{\text{SSP}}$ varies along a column of panels from first to sixth, while a row represents the results for SSP, CSSP, and VBCSSP. Colored bars are scaled for each row.

Figures 2 and 3 show SF and correlation of residuals. First, we explain the results for the SSP, which are presented in the left column in Figures 2 and 3. The SSP works most effectively when there is no gain error and no model misspecification, that is, 0% gain error with $L_{\text{true}}=L_{\text{SSP}}$ . The attenuation in shielding performance and correlation due to gain errors is more pronounced with a higher order of $L_{\text{SSP}}$ . In the case of $L_{\text{SSP}}=1$ , the shielding performance is attenuated by 5 [dB] with 5% gain errors. On the other hand, when $L_{\text{SSP}}=5$ , the shielding performance degrades by more than 10 [dB] with 5% gain errors. Similar tendencies are also confirmed by the correlation criterion. Thus, the higher the order of $L_{\text{SSP}}$ , the more important the channel gain estimation.

Next, we explain the results for the CSSP, which are presented in the middle column in Figures 2 and 3. In the case of $L_{\text{SSP}}=1$ , the magnitude of gain error does not affect performance. However, for large $L_{\text{SSP}}$ , as the magnitude of gain errors increases, both SF and CORR(e) significantly decrease.

Finally, we explain the results for VBCSSP, which are presented in the right column in Figures 2 and 3. For all $L_{\text{SSP}}$ , the magnitude of gain error does not affect performance. This suggests that VBCSSP robustly corrects gain errors for any $L_{\text{SSP}}$ .

For the SSP and CSSP with $L_{\text{SSP}}=6,$ SF becomes excessively large, but CORR(e) becomes nearly zero. This is due to the fact that the number of bases N=48 is larger than the number of channels *M=45* when $L_{\text{SSP}}=6$ , and thus the inverse matrix in Eq. (3) cannot be solved correctly. On the other hand, Eq. (14) by VBCSSP can be solved even in an under-determined situation due to the effect of the L2 normalization.

In the case of model misspecification ($L_{\text{true}}\neq L_{\text{SSP}}$ ), all methods work robustly when $L_{\text{SSP}}>L_{\text{true}}$ , but the opposite case is not confirmed. This is because a linear combination of lower-order bases cannot represent higher-order bases.

Summarizing the results in Figures 2 and 3, VBCSSP demonstrated greater robustness compared to SSP and CSSP, regardless of $L_{\text{SSP}}$ and gain error.

#### 4.1.2. Evaluating estimated channel gain and free energy

**Fig. 4.**
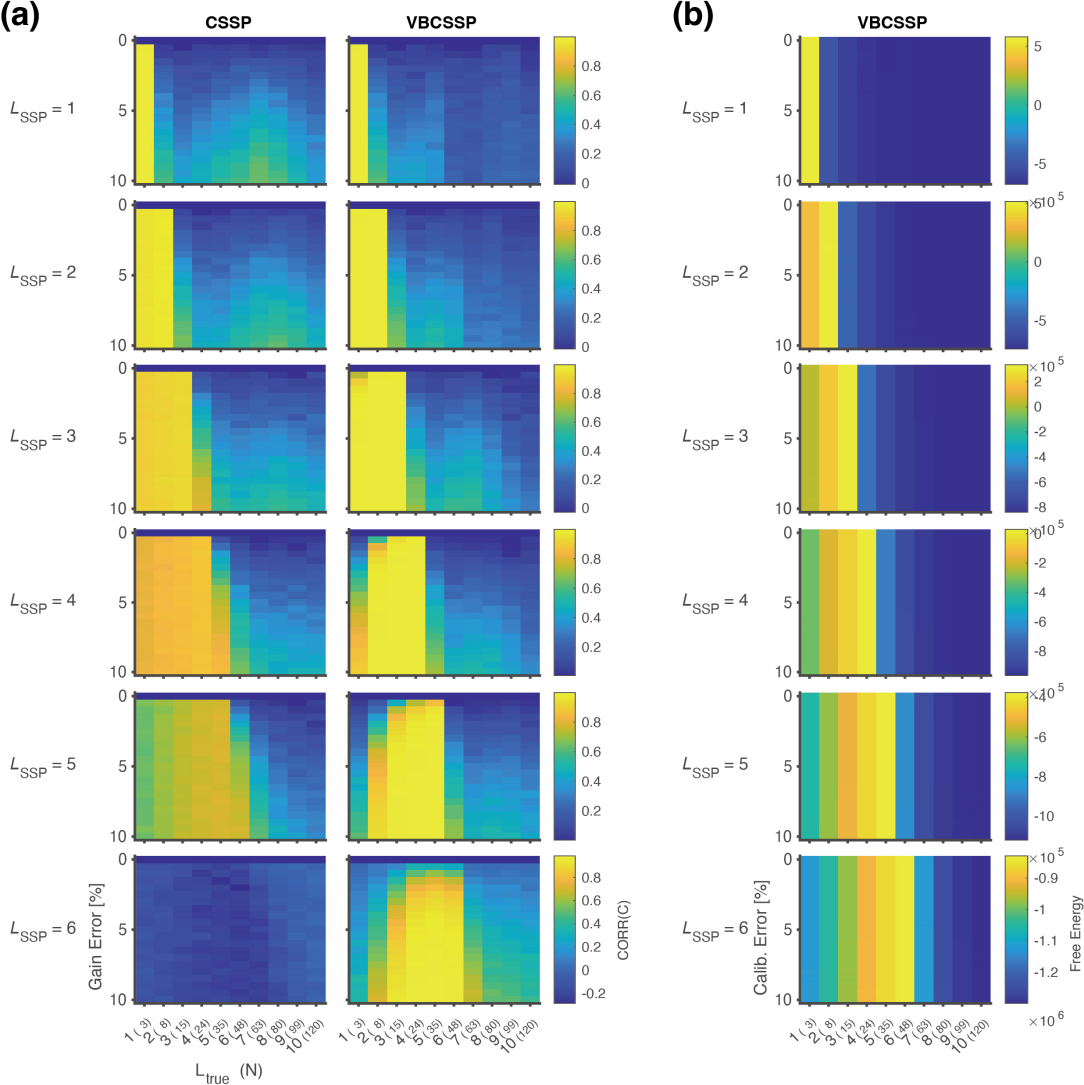
Accuracy of estimated channel gains (a) and free energy (b) for static simulation. (a) Correlation coefficient between true and estimated channel gains CORR(C). (b) Free energy of VBCSSP. In a panel, the horizontal axis is the order of the true model ${\text{L}}_{\text{true}}$ , and the vertical axis is the magnitude of gain error $100\sigma_{\text{C}}$ [%] for (a) or the free energy for (b). The number in parentheses on the horizontal axis represents the number of bases N for the true interference model ${\text{S}}_{\text{true}}$ , computed by $L_{true}^{2}+2L_{true}$ . ${\text{L}}_{\text{SSP}}$ varies along a column of panels from first to sixth, while a row represents the results for CSSP and VBCSSP. Colored bars are scaled for each row. Here, CORR(C) is not defined for any gain error (0 [%]) because variations of ${\text{C}}_{\text{true}}$ amount to 0.

Figure 4a shows the accuracy of channel gain estimated by CSSP and VBCSSP. In the case of CSSP, CORR(C) decreases by increasing $L_{\text{SSP}}$ . On the other hand, for VBCSSP, CORR(C) is stable for any $L_{\text{SSP}}$ . These results indicate that VBCSSP accurately estimated channel gain regardless of $L_{\text{SSP}}$ , while CSSP did not.

We also evaluated estimated free energy for VBCSSP in Figure 4b. The free energy is maximum when the SSP model matches the true model. This is valid for any gain error. This finding suggests the possibility that model selection is made on the basis of free energy.

#### 4.1.3. Evaluating lead field attenuation

**Fig. 5.**
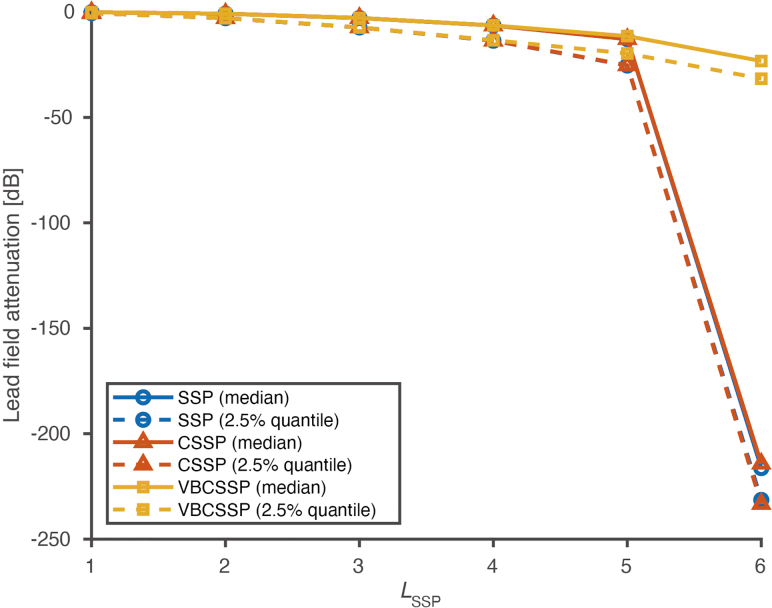
Lead field attenuation for static simulation. Of the $\text{attenuatio}{\text{n}}_{\text{i}}$ values computed for all cortical vertices i, only the median and 2.5% quantile are plotted for each method. The gain error was fixed at 10%. Note that we only visualized the results without model misspecification (i.e., ${\text{L}}_{\text{SSP}}={\text{L}}_{\text{true}}$ ).

We computed the lead field attenuation, $\;{\text{attenuation}}_{i}$, when the optimal model was used for each method (i.e., $L_{\text{SSP}}=L_{\text{true}}$ ). The gain error was fixed at 10%. Figure 5 shows the median and 2.5% quantile (i.e., vertex with particularly large attenuation) of all cortical vertices for SSP, CSSP, and VBCSSP.

VBCSSP demonstrated improvements in lead field attenuation over SSP and CSSP when $L_{\text{SSP}}\geq 5$ . This improvement was most pronounced at $L_{\text{SSP}}=6$ , where both SSP and CSSP estimates break down. At lower orders ($L_{\text{SSP}}\leq 4$ ), attenuations were comparable among all methods. In addition, the spatial distributions of attenuations were also consistent (spatial correlation > 0.99) among methods at lower orders. These results persisted regardless of the magnitude of gain error, even when no gain error was introduced (0%). Therefore, at lower orders, the effect of the VBCSSP projector on the lead field is not significantly different from that of SSP.

### 4.2 Dynamic channel gain simulation

**Fig. 6.**
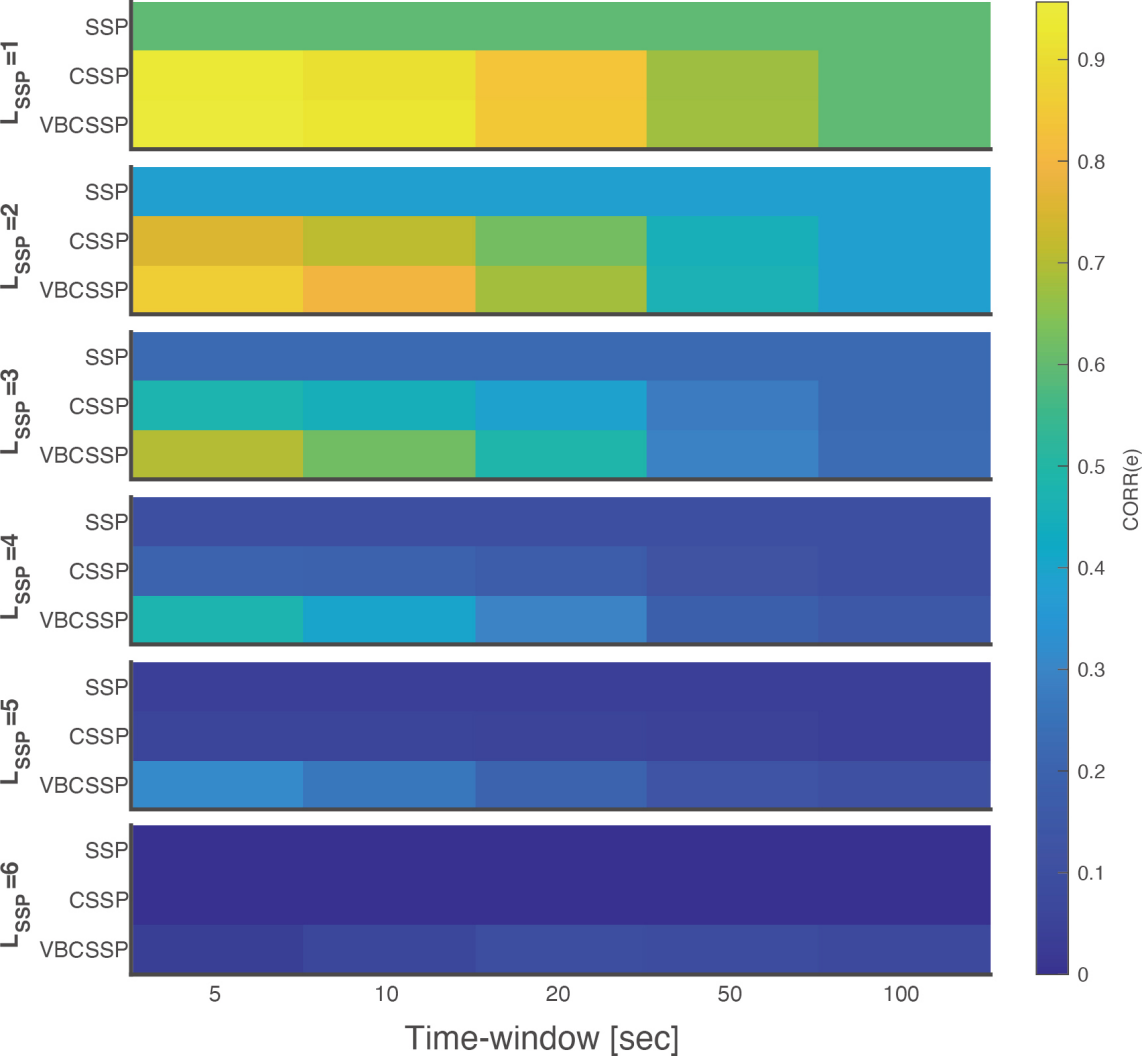
Similarity between true and estimated residuals for each time window. Each panel in the figure visualizes the correlation of residuals CORR(e) for each method with a specific order of spherical harmonics ${\text{L}}_{\text{SSP}}$ . The horizontal axis of a panel is the length of the time window, and the vertical axis represents the results for SSP, CSSP, and VBCSSP. ${\text{L}}_{\text{SSP}}$ varies along a column of panels from first to sixth. Note that we only visualized the results without model misspecification (i.e., ${\text{L}}_{\text{SSP}}={\text{L}}_{\text{true}}$ ).

**Fig. 7.**
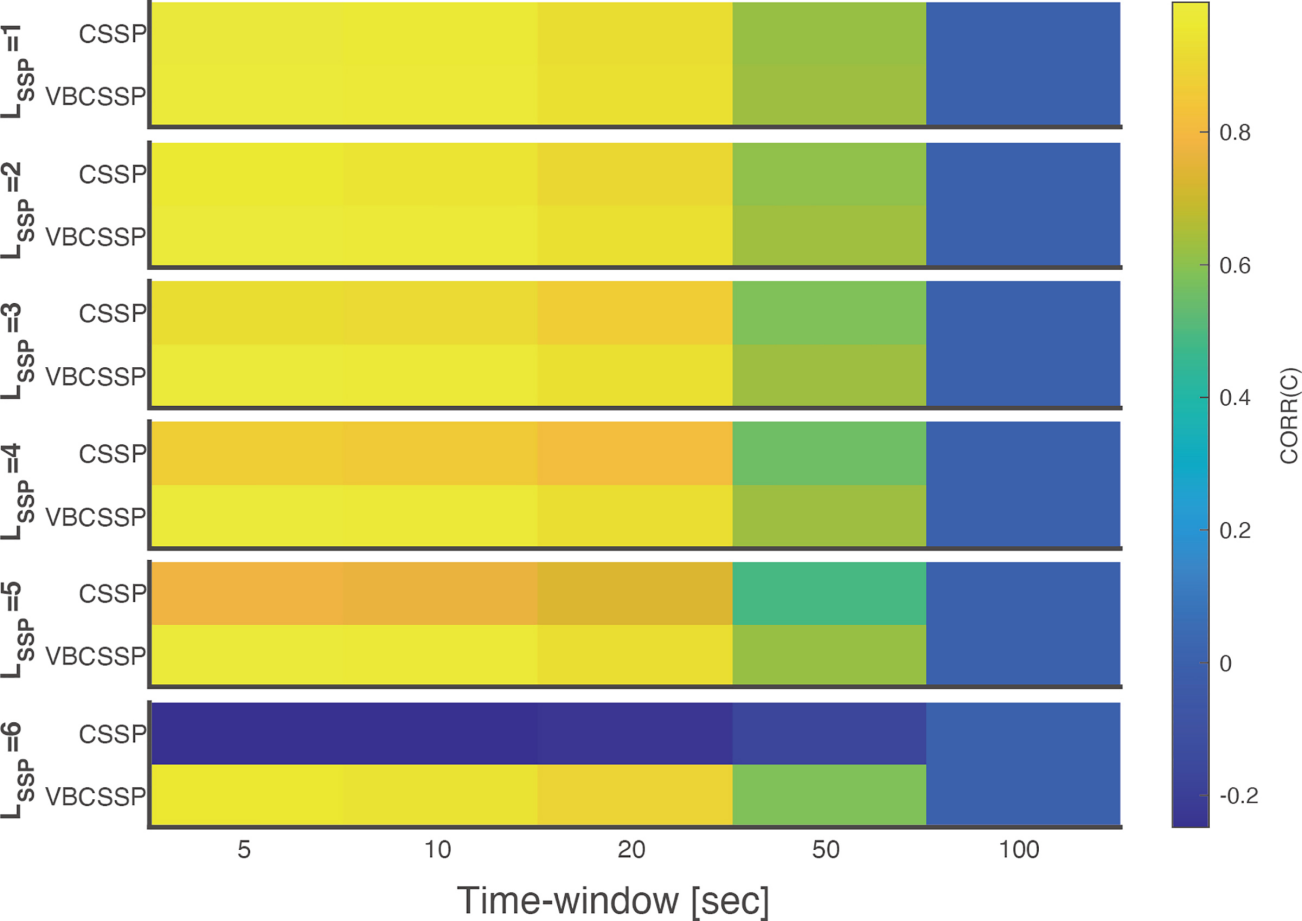
Accuracy of estimated channel gains for each time-window. Each panel in the figure visualizes the correlation coefficient between true and estimated channel gains CORR(C) for each method with a specific order of spherical harmonics ${\text{L}}_{\text{SSP}}$ . The horizontal axis of a panel is the length of the time window, and the vertical axis represents the results for CSSP and VBCSSP. ${\text{L}}_{\text{SSP}}$ varies along a column of panels from first to sixth. Note that we only visualized the results without model misspecification (i.e., ${\text{L}}_{\text{SSP}}={\text{L}}_{\text{true}}$ ).

Figure 6 shows that CORR(e) of SSP remains invariant to window length, since SSP depends solely on the interference model S and is unaffected by temporal input fluctuations. In contrast, both CSSP and VBCSSP yield higher CORR(e) than does SSP, with better performance observed as the window becomes shorter. Figure 7 also shows that CSSP and VBCSSP estimate the channel gain more accurately with shorter window lengths. These results stem from the fact that, within sufficiently short windows, C can be approximated as a quasi‑static value.

Focusing on the estimated C, VBCSSP consistently outperforms CSSP, as reflected in the CORR(e) results. Furthermore, for window lengths shorter than 10 sec, CORR(C) exceeds 0.9 regardless of $L_{\text{SSP}}$ . These findings suggest that, even in the presence of time-varying interference, VBCSSP can effectively track dynamic gain‑error fluctuations by segmenting the data into short windows.

### 4.3 MC experiment

**Fig. 8.**
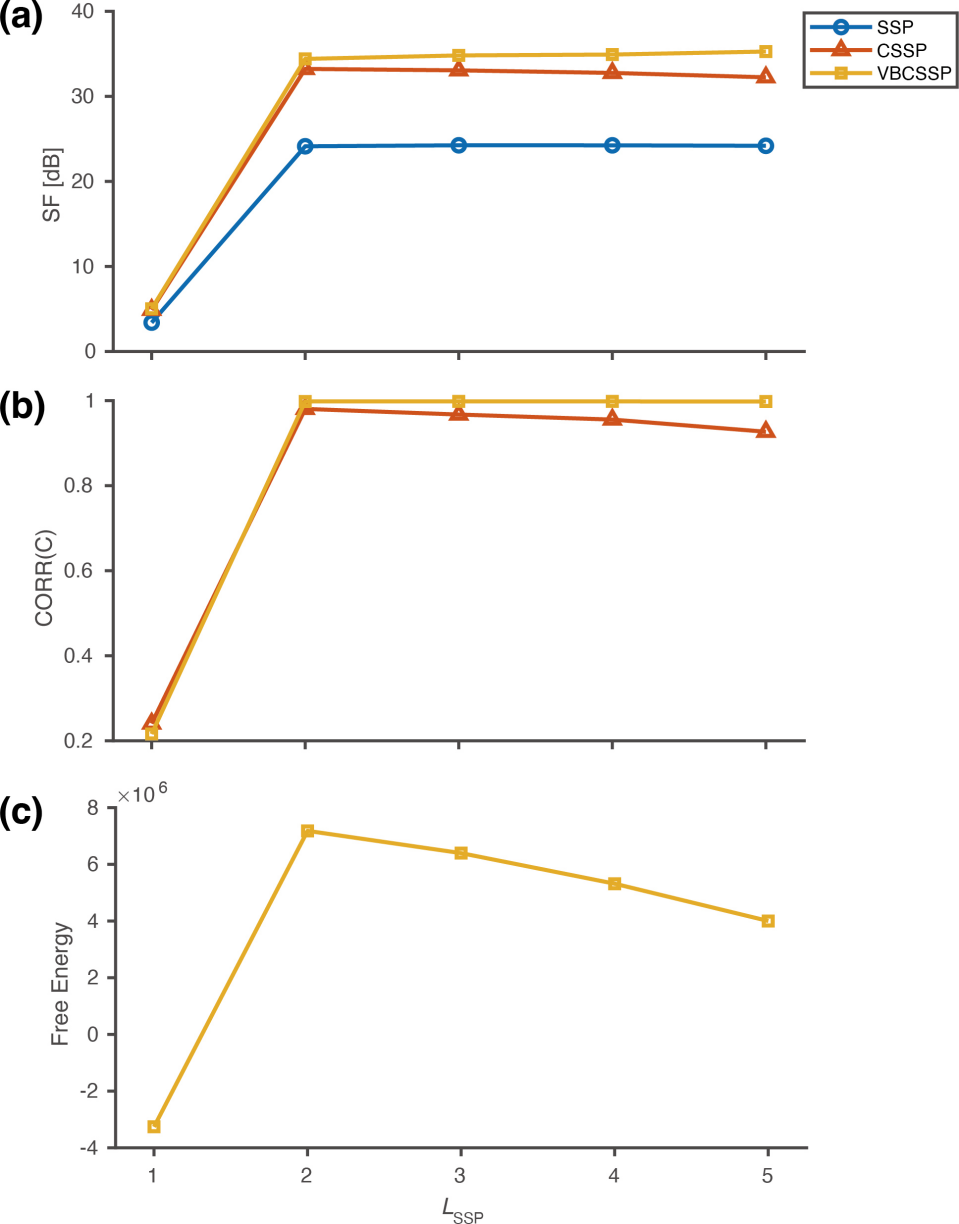
Shielding factor (a), Similarity of estimated channel gains (b), and Free energy (c) for MC experiment. In (a), (b) and (c), SF , CORR(C), and free energy was plotted as a function of ${\text{L}}_{\text{SSP}}$ , respectively. CORR(C) was computed by treating the channel gains estimated by the MC calibration process as the ground truth.

Figure 8 shows (a) SF , (b) CORR(C), and (c) free energy as a function of $L_{\text{SSP}}$ for each method. First, as shown in Figure 8a, SSP exhibits a markedly smaller SF compared to the other gain‑correction methods. Moreover, all methods reach a plateau once $L_{\text{SSP}}\geq 2$ , as expected due to the known harmonics in the dataset.

In terms of estimation accuracy of channel gains (Fig. 8b), VBCSSP outperforms CSSP, and this advantage becomes more pronounced as $L_{\text{SSP}}$ increases. CORR(C), improves markedly when $L_{\text{SSP}}$ changes from 1 to 2 but then declines slightly.

Finally, as shown in Figure 8c, free energy estimated by VBCSSP is maximal at $L_{\text{SSP}}=2$ . These results suggest that the interference field generated by the MC can be adequately described by the second‑order spherical harmonics function.

In summary, even in a real environment, VBCSSP not only has higher SF and more accurate C estimation capabilities than CSSP but also has the ability to select the optimal model.

### 4.4 Human experimental data

**Fig. 9.**
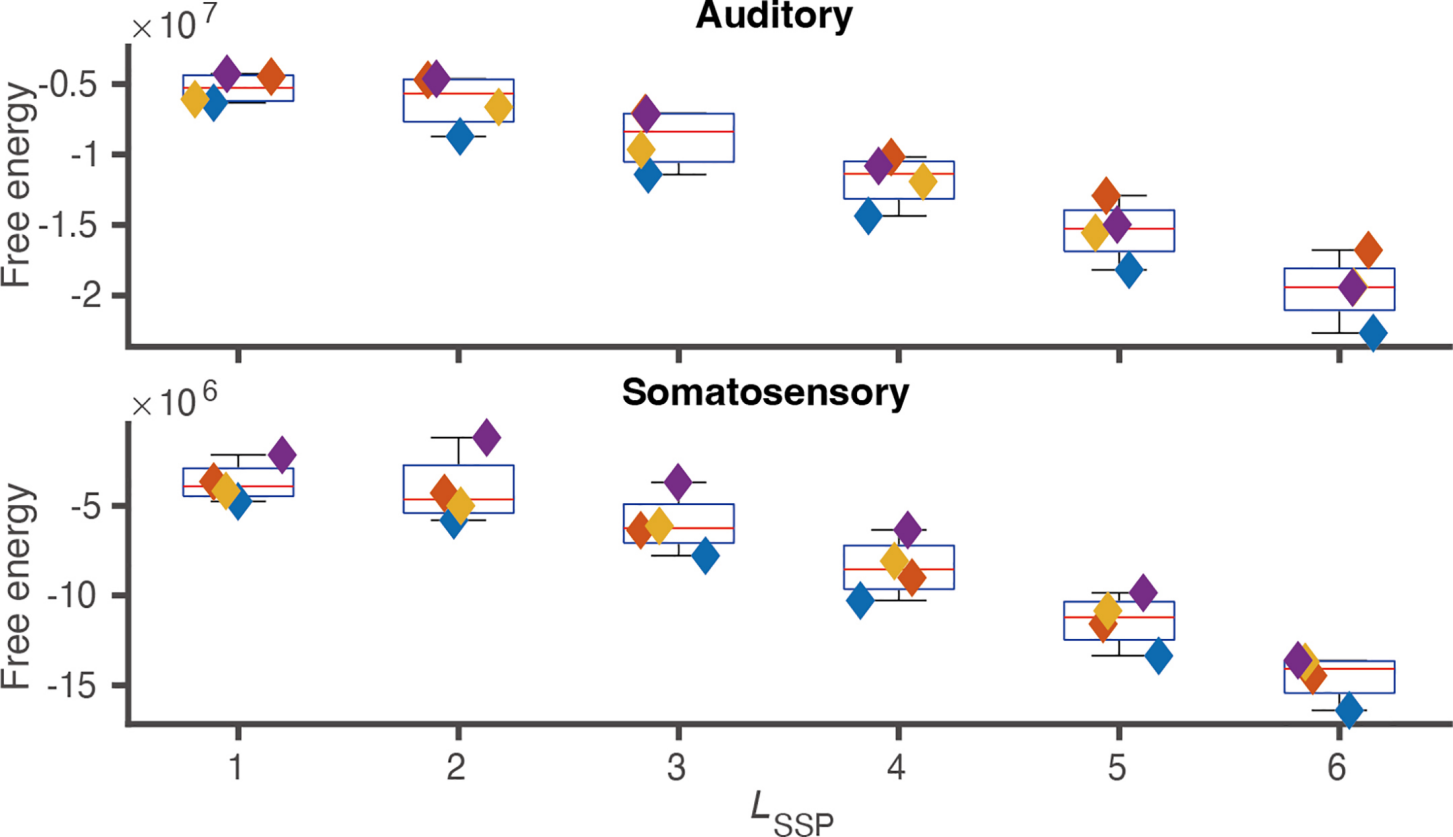
Free energy for human experimental data. The top and bottom panels show results for the auditory and somatosensory tasks. In each panel, the horizontal axis is the order of spherical harmonics ${\text{L}}_{\text{SSP}}$ , and the vertical axis is the free energy. Colored diamonds represent four different subjects.

We first attempted to select the optimal $L_{\text{SSP}}$ using the free energy criterion. We repeatedly performed VBCSSP using spherical harmonics bases with $L_{\text{SSP}}=1$ to 6 and then compared the free energy (Fig. 9). The free energy was relatively maximal for $L_{\text{SSP}}=1$ or 2 and decreased monotonically with increasing order. Based on these results, we performed SSP and VBCSSP with $L_{\text{SSP}}=1$ in the subsequent analysis. In applying VBCSSP, its algorithm (Algorithm 1) automatically rejected three or fewer channels around the occipital area. We show examples of estimated channel gain for auditory and somatosensory tasks in Supplementary Fig. S6.

**Fig. 10.**
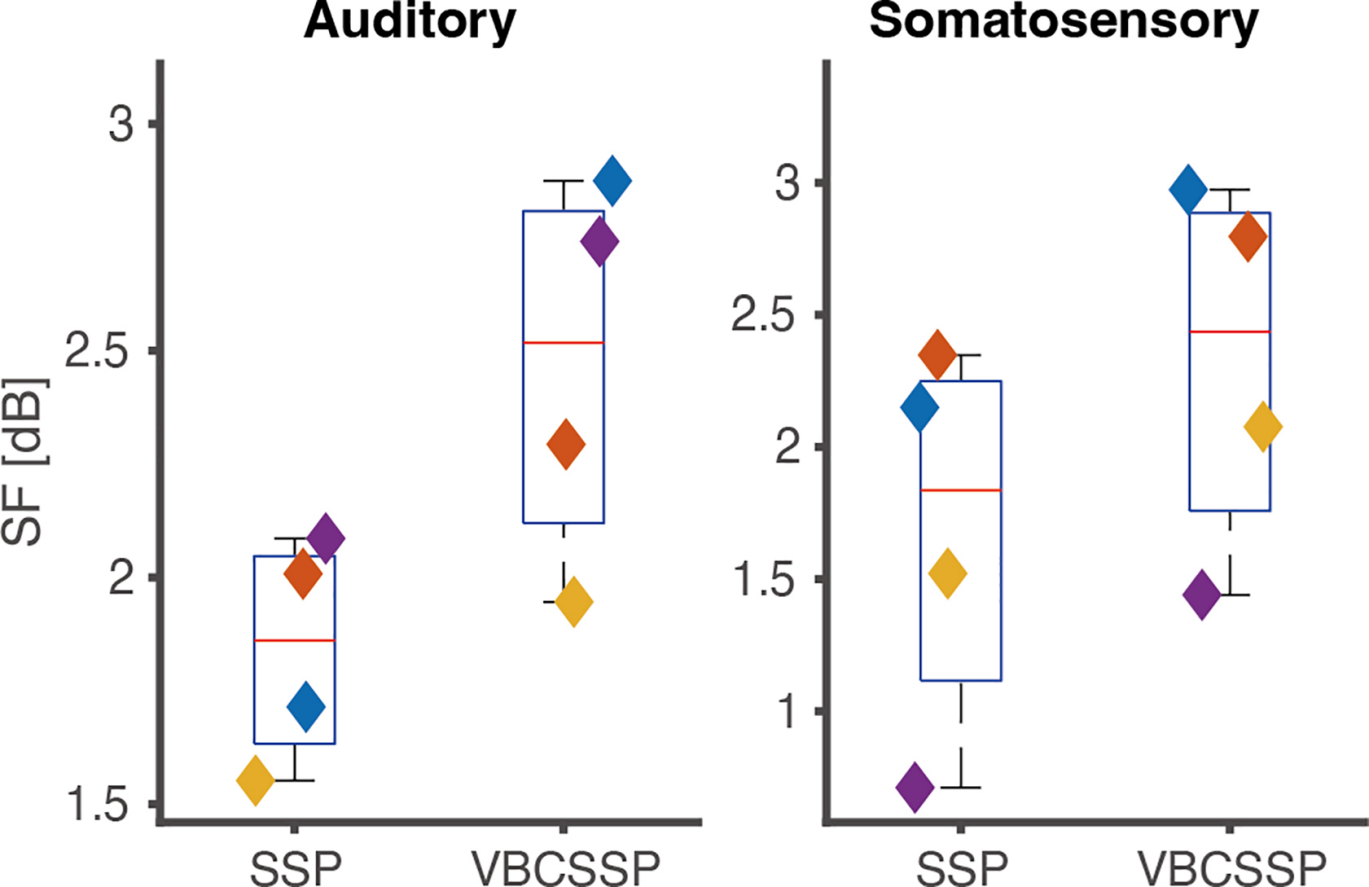
Shielding factor at 6–10 Hz for human experimental data. The left and right panels show the results for the auditory and somatosensory tasks. In each panel, the vertical axis is the shielding factor SF , which was computed by Eq. (24) and averaged at 6–10 Hz. Colored diamonds represent four different subjects.

Next, SSP and VBCSSP were compared using SF (Fig. 10). This was carried out using Eq. (24) for each frequency between 6 and 10 Hz and then averaging the values across the frequencies. Compared to SSP, VBCSSP improved SF by an average of 0.62 and 0.64 [dB] for the auditory and somatosensory tasks, respectively. This result suggests that VBCSSP removed a greater amount of interference field than SSP.

**Fig. 11.**
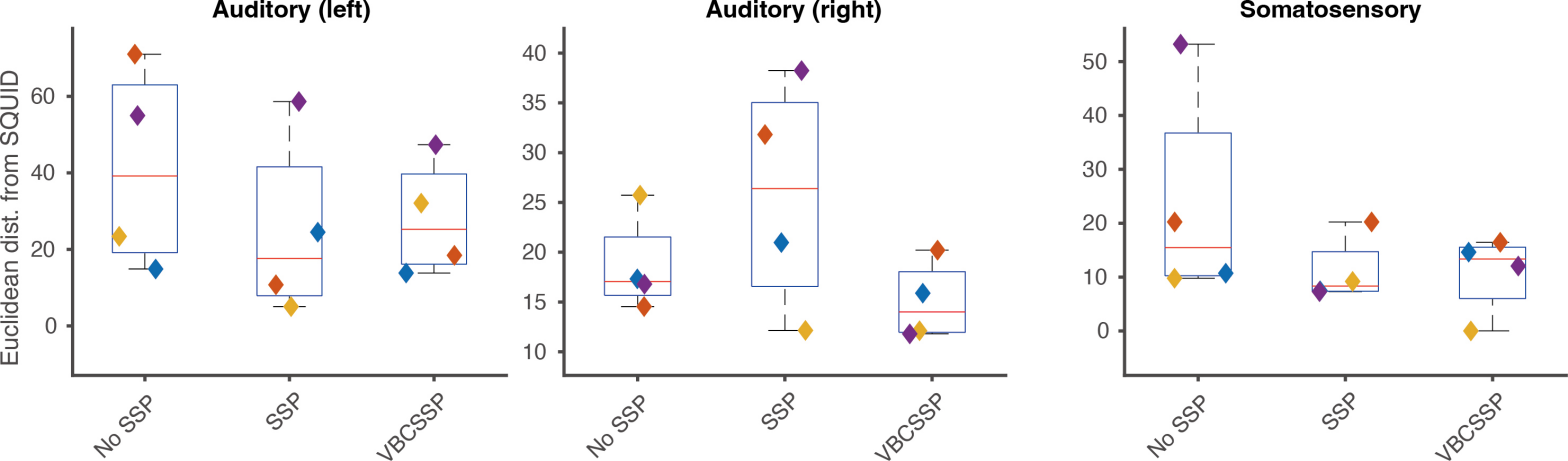
Proximity of estimated dipoles between SQUID-MEG and OPM for human experimental data. The left and middle panels show the results for each hemisphere of the auditory task. The right panel gives the results of the somatosensory task. In each panel, the vertical axis represents the Euclidean distance from the estimated dipole using SQUID-MEG data. In the case of “No SSP,” all preprocessing was performed except for SSPs. Colored diamonds represent four different subjects.

Finally, we evaluated dipoles reconstructed after preprocessing with SSP, with VBCSSP, and without any SSP (Fig. 11). In the somatosensory and auditory tasks, single-dipole fitting and double-dipole fitting (single dipole per hemisphere) were performed, respectively. For each preprocessing method, we computed the Euclidean distance from the reconstructed dipole from SQUID-MEG data. In the auditory (left) and somatosensory tasks, SSP and VBCSSP demonstrated shorter distances with smaller deviations than the results without SSP. In the auditory (right) case, SSP was worse than no SSP, whereas VBCSSP remained slightly better than or equivalent to the case of no SSP. These results suggest that VBCSSP did not have any negative effect on the source estimation.

## 5 Discussion

We proposed a variational Bayesian estimation method for channel gain calibrated SSP, which we validated through comprehensive evaluations on static and dynamic channel gain simulation, an MC experiment, and human experimental data. The results of the static simulation confirmed that VBCSSP successfully estimated non-uniform channel gain for any $L_{\text{SSP}}$ (Fig. 4). This is also reflected in the evaluation of SF (Fig. 2) and CORR(e) (Fig. 3). Compared to CSSP, which corrects for channel gain using least-square regression, the difference with VBCSSP becomes more pronounced as $L_{\text{SSP}}$ increases. This suggests the importance of iterative optimization of H and C, consistent with Iivanainen et al. (2022). In particular, CSSP cannot solve the under-determined case ($L_{\text{SSP}}=6$ ) because the least-square regression does not hold.

We assumed stationarity in the probability distributions of VBCSSP. However, the environmental magnetic field dynamically changes in real experiments due to large drift and the subject’s movement. An intrinsic solution to this problem is to minimize the drift by performing real-time feedback using the on-board coils (i.e., a closed-loop correction; Mellor et al., 2023; Robinson et al., 2022; Yan et al., 2022). This does not conflict with our method, and it can alleviate model violations. On the other hand, a post hoc method successfully improved the shielding performance for tasks involving large motion by performing spherical harmonics-based denoising for each of the small time windows (Mellor et al., 2022). Inspired by this study, we conducted dynamic gain simulation. We demonstrated that applying VBCSSP to short time windows enables effective adaptation to temporally varying gain errors (Figs. 6 and 7). This finding suggests that VBCSSP remains robust even when errors fluctuate due to low‑frequency drifts. We note that the minimum required window length depends on the temporal frequencies of interference that drive gain error, and excessively short windows do not always work well due to insufficient samples because shorter windows contain fewer sampling points, particularly after down-sampling.

Related to the above discussion, channel gain errors are categorized into static errors arising from inherent sensor characteristics and dynamic errors induced by low‑frequency drifts in the interference magnetic field. VBCSSP does not rely on any specific physical mechanism; rather, it simply adjusts each channel’s gain to optimally reconcile the observed magnetic field with the assumed interference model. Consequently, the types of errors corrected by VBCSSP depend heavily on the application context. Specifically, when VBCSSP is applied to all data points at once, temporal gain fluctuations are averaged out, yielding a correction of static calibration errors. In contrast, when applying VBCSSP on segmented data points, it tracks and corrects dynamically varying gain errors.

The closed-loop correction enables a dynamic range exceeding ± 100 nT and compensates for the cross-axis projection errors (Mellor et al., 2023; Robinson et al., 2022; Yan et al., 2022). However, static errors will likely remain an issue even if the influence of interference magnetic fields is completely eliminated. Our method complements the correction of each channel’s static characteristics when applied to signals corrected online by a closed-loop system.

Inspired by previous studies on model selection for observation noise (Lopez et al., 2019), sensor geometry (Duque-Muñoz et al., 2019), and the order of spherical harmonics (Iivanainen et al., 2022), we attempted to select the optimal order of spherical harmonics using the free energy criterion. Using the static simulation data, we confirmed that the correct orders were successfully determined based on free energy (Fig. 4). Moreover, in the MC experiment, SF and CORR(C) of VBCSSP reached a plateau at $L_{\text{SSP}}=2$ with the highest free energy, as expected due to the known harmonics in the dataset (Fig. 8). Interestingly, free energies varied smoothly along the orders. These suggest that the best model among candidates can be selected, providing a model selection method for practical situations.

We evaluated VBCSSP using the human experimental data. The key feature of our dataset is that it permits comparison with SQUID-MEG. We first decided the optimal $L_{\text{SSP}}$ using the free energy criterion (Fig. 9). Then, we confirmed that VBCSSP improved SF by about 0.6 [dB] compared to SSP (Fig. 10). Finally, the estimated dipoles’ agreement with SQUID-MEG was either comparable (auditory left, somatosensory) or improved by a few dozen millimeters (auditory right) (Fig. 11). Notably, VBCSSP did not have a negative impact on the estimation, while SSP could degrade the results when compared to the No-SSP case.

In the human experimental data, the above failure of SSP stems from a fundamental limitation of subspace-based methods. Specifically, when local activity that cannot be explained by the interference model propagates to all channels via projection, the performance degrades notably. In fact, substantial magnetic field gradients have been observed in our magnetically shielded room at ATR Neural Information Analysis Laboratories in Japan, where the human experimental data were recorded. Specifically, in the region occupied by the subject’s head during the experiment, the Euclidean norm of the five field gradient components—estimated by fitting the spherical harmonic field model (Rea et al., 2021)—was 53.4 nT/m. As demonstrated by Schoffelen et al. (2025), such gradients can induce large signal fluctuations in the nanotesla range, even with only slight head movements of only a few millimeters. VBCSSP addressed this issue by leveraging channel gains to suppress such locally suspicious channels, thus avoiding the failure of SSP. This is supported by the estimated gain map (Supplementary Fig. S6 in Supplementary Note 3), which shows locally large gains. Note that the consistent pattern across tasks can be attributed to the fact that the participant’s head position was almost stable due to placing it on the chin rest.

AMM have been reported to be particularly robust to calibration errors among the methods (Schoffelen et al., 2025; Tierney et al., 2024). Using another dataset (Rier et al., 2023) that has a sufficient number of channels for AMM, we compared the performance of VBCSSP with those of AMM and its temporal expansion (AMMt) (All compared methods are listed in Supplementary Table 1; further details are provided in Supplementary Note 2). For this, *L*_SSP_ was selected using the free energy of VBCSSP (Supplementary Fig. S2). AMM and AMMt demonstrated notably improved SF compared to SSP and VBCSSP, especially in the high-frequency bands (Supplementary Fig. S3). From the perspective of subject identifiability based on connectome, AMM showed a slight decrease in scores, while AMMt showed a slight improvement in scores (Supplementary Figs. S4 and S5). In conclusion, AMM is effective when the number of channels is sufficient, and by adopting temporal expansion, we expect it to enhance both SF and subject identifiability. On the other hand, from these perspectives, VBCSSP showed performance almost equivalent to that of SSP. However, due to its ability to estimate channel gain and free energy, VBCSSP has the potential to detect bad channels and to perform model selection. Therefore, VBCSSP can be used not only for signal preprocessing but also for model selection and bad channel detection, making it a useful complement to other methods such as AMM.

The MC experiment effectively supplemented our simulation experiments. Specifically, it confirms that VBCSSP operates reliably when realistic sensor characteristics (e.g., axis‑dependent noise level differences) are present. The results further suggest that VBCSSP could support hardware-based calibration protocols (Hill et al., 2025; Iivanainen et al., 2022; Liang et al., 2025; Pfeiffer et al., 2020). For example, in situations where the magnetic source can be controlled but its spatial distribution is unknown, employing VBCSSP for model selection may enable effective calibration.

In this study, we considered only spherical harmonics-based SSPs, since they are widely used for modeling interference subspaces due to their orthogonality and computational tractability. In practice, there are instances where the interference field cannot be adequately modeled by a lower-order spherical harmonics basis, such as in the presence of a localized noise source. If a particular noise source were modeled mathematically, it could be employed as an interference model of VBCSSP. In such cases, the free energy might be used to evaluate evidence for using the model. Furthermore, if multiple candidates were possible, it might be possible to select the best model based on free energy.

Figures 2 and 3 show that all methods are robust when $L_{\text{SSP}}$ is larger than $L_{\text{true}}$ , but the converse was not confirmed. On the other hand, Figure 3 also shows that the maximum CORR(e) decreases as $L_{\text{SSP}}$ increases. These results imply that higher-order models harm the post-projection signal, even though a higher $L_{\text{SSP}}$ is required to suppress high spatial frequency interference. Therefore, the ideal operation would be to perform the lowest-order VBCSSP in a homogeneous interference field. This reinforces the importance of controlling the remnant field. It is also important to reduce the order of interference by preprocessing techniques such as filtering or ICA.

In the results of CORR(e) (Fig. 3), the maximum value of the color bar decreases with higher $L_{\text{SSP}}$ . In the case of $L_{\text{SSP}}=5$ , the maximum correlation coefficient is about 0.45 (Fig. 3). This shows a major limitation of SSP-based methods, that is, that suppressing higher-order interference is difficult even without model misspecification. This issue arises because, at higher harmonic orders, the estimation of H becomes increasingly ill‑conditioned. Moreover, the overlap between the neural signal subspace (e.g., the lead‑field or irregular spherical harmonics) and the interference subspace (high‑order regular spherical harmonics) continues to grow. In effect, components of the neural subspace can eventually be explained by the interference basis contaminating H and the residual term, making it challenging to accurately estimate them.

Negative gain estimates can be explained by a channel whose polarity is inverted relative to its surrounding channels under the modeled field. Such inversion can occur, for example, when a tangential axis of an OPM sensor is flipped in its holder while the radial axis remains correctly oriented. In the dataset of Supplementary Note 2, we observed this pattern for Subject 010: two rejected channels corresponded to the tangential axes of the same sensor. While this interpretation for Subject 010 is speculative, it represents a plausible measurement error in the actual experiment because OPM systems require many sensors to be placed manually in a head cap, which can lead to occasional misplacement. In such cases, our method could assist in identifying misplaced channels in post hoc analysis.

All sensors adopted in this study were QZFM units from QuSpin (https://quspin.com), spatially arranged by custom configurations. However, commercially available alternatives—for example, FieldLine’s HEDscan system (https://fieldlineinc.com) and MAG4Health’s helium-based sensors (https://www.mag4health.com)—are also widely used. Because VBCSSP makes no assumption on specific physical mechanisms, it should be equally effective with helium‑based sensors. On the other hand, although VBCSSP is agnostic to sensor layout, it is important to sample a greater amount of interference subspace to estimate a more accurate H. In particular, for dual‑axis sensors, the relative orientation of the tangential axes is important. For example, FieldLine’s HEDscan alternates its tangential axes by 90 degrees between adjacent channels, providing an effective strategy for sampling the interference subspace (Schoffelen et al., 2025).

Since the estimated values of H and C are mutually adjustable, their absolute values are meaningless. However, by applying correction using the norm of the SSP solution (Eq. (15)), we confirmed that this indeterminacy is effectively mitigated for $L_{\text{SSP}}\leq 3$ (see Supplementary Note 1). Because interference with fourth-order or higher cases is quite rare in a magnetically shielded room, this indeterminacy is unlikely to pose a problem under realistic conditions. On the other hand, we need to account for the influence of the chosen interference model S on the estimates. In other words, the estimated H and C are only valid under the assumed model S. Since the true interference model is unknown in real situations, the interpretations of H and C must be made with caution.

VBCSSP Eq. (4) assumes that matrix C is involved only in the interference term, not in the residual term containing neural signals. This simplification is based on the assumption that neural signals can be ignored for the purpose of estimating C, since they are orders of magnitude smaller than interference signals. Some readers, however, may wish to incorporate an explicit neural-signal model into both S and H**,** as in Tierney et al. (2022). However, as demonstrated in Supplementary Note 1, the indeterminacy between H and C can only be reliably resolved at low model orders ($L_{\text{SSP}}\leq 3$ ). Including high-order neural components (e.g., ninth-order spherical harmonics, which comprise 99 bases) in the model would, thus, be impractical. Consequently, extending VBCSSP to the SSS framework is challenging.

Appropriate channel calibration has been reported to improve subsequent analyses, such as current source reconstruction (Hill et al., 2025). As demonstrated by the simulation results and the MC experiment, the estimated C closely matches the true or MC-estimated channel gains. Therefore, our method is expected to enhance subsequent analyses by using the estimated C to correct the channel gains of neural signals.

Few-channel systems are not only used for cortical MEG (often in a custom-built prototype system) but are also found in biomagnetic measurements using OPMs, namely spinal recordings (Mardell et al., 2024), peripheral nerve recordings (Bu et al., 2022), cardiography (Strand et al., 2019), and myography (Marquetand et al., 2021). In these systems, modeling the magnetic field of interest is not well established, making the application of SSS-based methods difficult. VBCSSP is expected to operate robustly under these applications because it works with few-channel systems without assuming signal subspace. This capability should greatly expand the future possibilities of OPM.

Our method estimates only the diagonal part of the calibration matrix C in Eq. (4). When C is a diagonal matrix, the C-step Eq. (10) remains well-posed computation. However, if C is a full matrix, additional restrictions, such as sparsity, are required to obtain a stable solution. In this case, interpreting the estimated off-diagonal elements becomes challenging. Here, a practical solution would be to focus on the off-diagonal elements between channels within the same sensor. This would enable us to compensate for cross-axis projection errors while maintaining stability and interpretability in the model.

## Supplementary Material

Supplementary Material

## Data Availability

In this study, we employed two publicly available datasets (https://vbmeg.atr.jp/nictitaku209/; Rier et al., 2023). In addition, the code for the VBCSSP algorithm can be downloaded (https://github.com/keitasuzu/vb_cssp/tree/main).
