# Supplementary material for: Channel gain calibrated signal space projection using variational Bayesian estimation for optically pumped magnetometers
Source: Imaging Neurosci (Camb). 2026 Feb 18;4:IMAG.a.1137. doi: 10.1162/IMAG.a.1137 (PMC12918756; doi:10.1162/IMAG.a.1137)
Supplement: Supplementary Material [file IMAG.a.1137_supp.pdf]

Supplementary Mathematical Derivations A. Deriving variational Bayesian EM algorithm

Joint posterior distribution (Eq. (8)) cannot be solved analytically; instead, we consider the free energy to derive the posterior distributions:

$$\mathcal{F} = \iint Q(\boldsymbol{H}|\boldsymbol{B})Q(\boldsymbol{c}_d|\boldsymbol{B}) \log \frac{P(\boldsymbol{H}, \boldsymbol{c}_d, \boldsymbol{B})}{Q(\boldsymbol{H}, \boldsymbol{c}_d|\boldsymbol{B})} d\boldsymbol{h} d\boldsymbol{c}_d \,. \qquad \text{(S1)}$$

Here, maximizing the free energy guarantees maximizing the lower bound of the evidence $P(\boldsymbol{B})$ (Bishop, 2006). In the above, the denominator is represented by the following two variational posterior distributions due to variational approximation:

$$Q(\boldsymbol{H}, \boldsymbol{c}_d|\boldsymbol{B}) = Q(\boldsymbol{H}|\boldsymbol{B})Q(\boldsymbol{c}_d|\boldsymbol{B}) \,. \qquad \text{(S2)}$$

Under the Gaussian model assumption (Eqs. (5), (6), and (7)), the above posteriors of $\boldsymbol{h}$ and $\boldsymbol{c}_d$ become the following Gaussian distributions due to conjugacy:

$$\begin{aligned} Q(\boldsymbol{H}|\boldsymbol{B}) &= Q\big(\boldsymbol{h}(1), \dots, \boldsymbol{h}(T) | \boldsymbol{b}(1), \dots, \boldsymbol{b}(T)\big), \\ &= \prod_{t=1}^{T} \mathcal{N}\big(\boldsymbol{h}(t) | \bar{\boldsymbol{h}}(t), \boldsymbol{\Gamma}^{-1}\big) \end{aligned} \qquad \text{(S3)}$$

and

$$Q(\boldsymbol{c}_d|\boldsymbol{B}) = \mathcal{N}(\boldsymbol{c}_d|\overline{\boldsymbol{c}_d}, \boldsymbol{\Phi}^{-1}) \,, \qquad \text{(S4)}$$

where $\boldsymbol{c}_d = (c_1, \dots, c_M)^{\top}$ is the vectorized diagonal part of $\boldsymbol{C}$, and $\boldsymbol{\Gamma}$ and $\boldsymbol{\Phi}$ are the precision matrices of posterior for $\boldsymbol{h}$ and $\boldsymbol{c}_d$, respectively. Then, we derive $Q(\boldsymbol{H}|\boldsymbol{B})$ and $Q(\boldsymbol{c}_d|\boldsymbol{B})$ as the distributions that maximize free energy $\mathcal{F}$. This maximization is achieved as a sequential optimization for $Q(\boldsymbol{H}|\boldsymbol{B})$ and $Q(\boldsymbol{c}_d|\boldsymbol{B})$, which are derived as the following H- and C-steps, respectively.

H-step

In this step, $Q(\boldsymbol{H}|\boldsymbol{B})$ is obtained by taking the expectation of the joint posterior $P(\boldsymbol{H}, \boldsymbol{c}_d, \boldsymbol{B})$ for $Q(\boldsymbol{c}_d|\boldsymbol{B})$ (Sekihara & Nagarajan, 2015):

$$\begin{aligned} \log Q(\boldsymbol{H}|\boldsymbol{B}) &= \langle \log P(\boldsymbol{H}, \boldsymbol{c}_d, \boldsymbol{B}) \rangle, \\ &= \sum_{t=1}^{T} \langle \log \mathcal{N}(\boldsymbol{b}(t)|\boldsymbol{C}\boldsymbol{S}\boldsymbol{h}(t), \boldsymbol{\Lambda}^{-1}) + \log \mathcal{N}(\boldsymbol{h}(t)|\boldsymbol{0}, \boldsymbol{I}) \rangle + \xi \,. \end{aligned} \qquad \text{(S5)}$$

In this step, $\langle\ \ \rangle$ indicates the expectation for $Q(\boldsymbol{c}_d|\boldsymbol{B})$, and ξ indicates terms unrelated to differentiation by $\boldsymbol{h}(t)$. Since the posterior distributions are assumed to be independent at each time point, we focus on the equation at time t:

$$\begin{aligned}&\langle\log\mathcal{N}\left(\boldsymbol{b}(t)|\boldsymbol{CSh}(t),\boldsymbol{\Lambda}^{-1}\right)+\log\mathcal{N}(\boldsymbol{h}(t)|\boldsymbol{0},\boldsymbol{I})\rangle\\&=-\frac{1}{2}\langle\{\boldsymbol{b}(t)-\boldsymbol{CSh}(t)\}^{\top}\boldsymbol{\Lambda}\{\boldsymbol{b}(t)-\boldsymbol{CSh}(t)\}\\&+\boldsymbol{h}^{\top}(t)\boldsymbol{h}(t)\rangle\,.\end{aligned}\tag{S6}$$

Here, the mean of the posterior is equal to the maximum of Eq. (S6) due to the Gaussian distribution. Each term on the right-hand side of Eq. (S6) is differentiated as follows:

$$\frac{\partial}{\partial\boldsymbol{h}(t)}\{\boldsymbol{b}(t)-\boldsymbol{CSh}(t)\}^{\top}\boldsymbol{\Lambda}\{\boldsymbol{b}(t)-\boldsymbol{CSh}(t)\}=-2\boldsymbol{CS}^{\top}\boldsymbol{\Lambda}\{\boldsymbol{b}(t)-\boldsymbol{CSh}(t)\}\tag{S7}$$

and

$$\frac{\partial}{\partial\boldsymbol{h}(t)}\boldsymbol{h}^{\top}(t)\boldsymbol{h}(t)=2\boldsymbol{h}(t)\,.\tag{S8}$$

Substituting Eqs. (S7) and (S8) to the differentiation of Eq. (S6), we obtain

$$\begin{aligned}&\frac{\partial}{\partial\boldsymbol{h}(t)}\log Q(\boldsymbol{h}(t)|\boldsymbol{b}(t))=\langle\boldsymbol{S}^{\top}\boldsymbol{C\Lambda b}(t)-\boldsymbol{S}^{\top}\boldsymbol{C\Lambda CSh}(t)\rangle-\boldsymbol{h}(t)\,,\\&=\boldsymbol{S}^{\top}\overline{\boldsymbol{C}}\boldsymbol{\Lambda b}(t)-\boldsymbol{S}^{\top}\overline{\boldsymbol{C}^2}\boldsymbol{\Lambda Sh}(t)-\boldsymbol{h}(t)\,.\end{aligned}\tag{S9}$$

Putting 0 on the left side, we obtain the mean of the posterior distribution in Eq. (S3):

$$\overline{\boldsymbol{h}}(t)=\left\{\boldsymbol{S}^{\top}\overline{\boldsymbol{C}^2}\boldsymbol{\Lambda S}+\boldsymbol{I}\right\}^{-1}\boldsymbol{S}^{\top}\overline{\boldsymbol{C}}\boldsymbol{\Lambda b}(t)\,.\tag{S10}$$

Finally, we derive Eq. (9) by substituting $\boldsymbol{\Gamma}$ in Eq. (11) to Eq. (S10).
Note that the expectation for $\boldsymbol{C}$ is calculated as

$$\begin{aligned}\overline{\boldsymbol{C}}&=\langle\mathrm{diag}(\boldsymbol{c}_d)\rangle\,,\\&=\mathrm{diag}(\overline{\boldsymbol{c}_d})\,,\end{aligned}\tag{S11}$$

and

$$
\begin{aligned}
\overline{\boldsymbol{C}^2} &= \langle \mathrm{diag}(\boldsymbol{c}_d)^2 \rangle\,, \\
&= \mathrm{diag}(\langle \boldsymbol{c}_d \boldsymbol{c}_d^{\top} \rangle)\,, \\
&= \mathrm{diag}(\boldsymbol{\Phi}^{-1} + \langle \boldsymbol{c}_d \rangle \langle \boldsymbol{c}_d \rangle^{\top})\,, \\
&= \mathrm{diag}(\boldsymbol{\Phi}^{-1}) + \left(\overline{c_1}^2, \dots, \overline{c_M}^2\right)^{\top},
\end{aligned} \tag{S12}
$$

where the diag function indicates "taking diagonal part" for matrix and "composing diagonal matrix" for vector, and $\overline{c_m}$ are the elements of $\overline{\boldsymbol{c}_d}$.

C-step

In this step, $Q(\boldsymbol{c}_d|\boldsymbol{B})$ is obtained by taking the expectation of the joint posterior $P(\boldsymbol{H}, \boldsymbol{c}_d, \boldsymbol{B})$ for $Q(\boldsymbol{H}|\boldsymbol{B})$:

$$
\begin{aligned}
\log Q(\boldsymbol{c}_d|\boldsymbol{B}) &= \langle \log P(\boldsymbol{H}, \boldsymbol{c}_d, \boldsymbol{B}) \rangle\,, \\
&= \left\langle \sum_{t=1}^{T} \log \mathcal{N}(\boldsymbol{b}(t)|\boldsymbol{CSh}(t), \boldsymbol{\Lambda}^{-1}) + \log \mathcal{N}(\boldsymbol{c}_d|\boldsymbol{0}, \boldsymbol{D}^{-1}) \right\rangle + \xi\,, \\
&= -\frac{1}{2}\sum_{t=1}^{T} \langle \{\boldsymbol{b}(t) - \boldsymbol{CSh}(t)\}^{\top} \boldsymbol{\Lambda} \{\boldsymbol{b}(t) - \boldsymbol{CSh}(t)\} \rangle - \frac{1}{2}\boldsymbol{c}_d^{\top} \boldsymbol{D} \boldsymbol{c}_d + \xi\,.
\end{aligned} \tag{S13}
$$

In this step, $\langle\ \rangle$ indicates the expectation for $Q(\boldsymbol{H}|\boldsymbol{B})$, and ξ indicates terms unrelated to differentiation by $\boldsymbol{c}_d$. Each term on the right-hand side of Eq. (S13) is differentiated as follows:

$$
\begin{aligned}
\frac{\partial}{\partial \boldsymbol{c}_d}\left\{-\frac{1}{2}\boldsymbol{c}_d^{\top} \boldsymbol{D} \boldsymbol{c}_d\right\} &= -\frac{1}{2}\left(\boldsymbol{D} + \boldsymbol{D}^{\top}\right)\boldsymbol{c}_d\,, \\
&= -\boldsymbol{D}\boldsymbol{c}_d\,,
\end{aligned} \tag{S14}
$$

and

$$\frac{\partial}{\partial \boldsymbol{C}}\left\{-\frac{1}{2}\sum_{t=1}^{T}\langle\{\boldsymbol{b}(t)-\boldsymbol{CSh}(t)\}^{\top}\boldsymbol{\Lambda}\{\boldsymbol{b}(t)-\boldsymbol{CSh}(t)\}\rangle\right\}$$

$$=-\frac{1}{2}\sum_{t=1}^{T}\frac{\partial}{\partial \boldsymbol{C}}\langle-2\mathrm{tr}\left[\boldsymbol{Sh}(t)\boldsymbol{b}^{\top}(t)\boldsymbol{\Lambda C}\right] \tag{S15}$$

$$+\mathrm{tr}\left[\boldsymbol{Sh}(t)\boldsymbol{h}^{\top}(t)\boldsymbol{S}^{\top}\boldsymbol{\Lambda C}^{2}\right]\rangle .$$

For any diagonal matrix $\boldsymbol{X}$, the following holds:

$$\frac{\partial}{\partial \boldsymbol{X}}\mathrm{tr}[\boldsymbol{AX}]=\boldsymbol{A}\circ\boldsymbol{I},$$
$$\frac{\partial}{\partial \boldsymbol{X}}\mathrm{tr}\left[\boldsymbol{AX}^{2}\right]=2\boldsymbol{A}\circ\boldsymbol{X}, \tag{S16}$$

where $\circ$ indicates Hadamard product. By applying Eq. (S16) to each term of (S15), the right-hand side becomes

$$\frac{\partial}{\partial \boldsymbol{C}}\mathrm{tr}\left[\boldsymbol{Sh}(t)\boldsymbol{b}^{\top}(t)\boldsymbol{\Lambda C}\right]=\left(\boldsymbol{Sh}(t)\boldsymbol{b}^{\top}(t)\boldsymbol{\Lambda}\right)\circ\boldsymbol{I}_{M},$$
$$\frac{\partial}{\partial \boldsymbol{C}}\mathrm{tr}\left[\boldsymbol{Sh}(t)\boldsymbol{h}^{\top}(t)\boldsymbol{S}^{\top}\boldsymbol{\Lambda C}^{2}\right]=2\left(\boldsymbol{Sh}(t)\boldsymbol{h}^{\top}(t)\boldsymbol{S}^{\top}\boldsymbol{\Lambda}\right)\circ\boldsymbol{C}. \tag{S17}$$

By substituting Eq. (S17) to (S15), we obtain

$$(\mathrm{S15})=-\frac{1}{2}\sum_{t=1}^{T}\langle-2(\boldsymbol{Sh}(t)\boldsymbol{b}^{\top}(t)\boldsymbol{\Lambda})\circ\boldsymbol{I}_{M}+2(\boldsymbol{Sh}(t)\boldsymbol{h}^{\top}(t)\boldsymbol{S}^{\top}\boldsymbol{\Lambda})\circ\boldsymbol{C}\rangle,$$

$$=\left\{\boldsymbol{S}\sum_{t=1}^{T}\left(\langle\boldsymbol{h}(t)\rangle\boldsymbol{b}^{\top}(t)\right)\boldsymbol{\Lambda}\right\}\circ\boldsymbol{I}_{M}-\left\{\boldsymbol{S}\sum_{t=1}^{T}\langle\boldsymbol{h}(t)\boldsymbol{h}^{\top}(t)\rangle\boldsymbol{S}^{\top}\boldsymbol{\Lambda}\right\}\circ\boldsymbol{C}, \tag{S18}$$

$$=\left\{\boldsymbol{S}\sum_{t=1}^{T}\left(\bar{\boldsymbol{h}}(t)\boldsymbol{b}^{\top}(t)\right)\boldsymbol{\Lambda}\right\}\circ\boldsymbol{I}_{M}-\left\{\boldsymbol{S}\sum_{t=1}^{T}\left(\boldsymbol{\Gamma}^{-1}+\bar{\boldsymbol{h}}(t)\bar{\boldsymbol{h}}^{\top}(t)\right)\boldsymbol{S}^{\top}\boldsymbol{\Lambda}\right\}\circ\boldsymbol{C},$$

$$=\{\boldsymbol{SR}_{hb}\boldsymbol{\Lambda}\}\circ\boldsymbol{I}_{M}-\{\boldsymbol{SR}_{hh}\boldsymbol{S}^{\top}\boldsymbol{\Lambda}\}\circ\boldsymbol{C},$$

where $\boldsymbol{R}_{hb}=\sum\left(\bar{\boldsymbol{h}}(t)\boldsymbol{b}^{\top}(t)\right)$ and $\boldsymbol{R}_{hh}=T\boldsymbol{\Gamma}^{-1}+\sum\left(\bar{\boldsymbol{h}}(t)\bar{\boldsymbol{h}}^{\top}(t)\right)$.

Note that the Hadamard product with a diagonal matrix becomes a diagonal matrix. Therefore, the differentiation of the first term in Eq. (S13) with respect to $\boldsymbol{c}_d$ can be expressed as

$$\frac{\partial}{\partial \boldsymbol{c}_d}\left\{-\frac{1}{2}\sum_{t=1}^{T}\langle\{\boldsymbol{b}(t)-\boldsymbol{CSh}(t)\}^{\top}\boldsymbol{\Lambda}\{\boldsymbol{b}(t)-\boldsymbol{CSh}(t)\}\rangle\right\}$$
$$=\mathrm{diag}(\boldsymbol{SR}_{hb}\boldsymbol{\Lambda})-\mathrm{diag}\left(\boldsymbol{SR}_{hh}\boldsymbol{S}^{\top}\boldsymbol{\Lambda}\right)\circ\boldsymbol{c}_d\,. \tag{S19}$$

Substituting Eq. (S14) and (S19) to the differentiation of Eq. (S13), we obtain

$$\frac{\partial}{\partial \boldsymbol{c}_d}\log Q(\boldsymbol{c}_d|\boldsymbol{B})=\mathrm{diag}(\boldsymbol{SR}_{hb}\boldsymbol{\Lambda})-\mathrm{diag}\left(\boldsymbol{SR}_{hh}\boldsymbol{S}^{\top}\boldsymbol{\Lambda}\right)\circ\boldsymbol{c}_d-\boldsymbol{D}\boldsymbol{c}_d\,. \tag{S20}$$

Putting 0 on the left side, we obtain the mean of posterior distribution for Eq. (S4):

$$\mathrm{diag}\left(\boldsymbol{SR}_{hh}\boldsymbol{S}^{\top}\boldsymbol{\Lambda}\right)\circ\overline{\boldsymbol{c}_d}+\boldsymbol{D}\overline{\boldsymbol{c}_d}=\mathrm{diag}(\boldsymbol{SR}_{hb}\boldsymbol{\Lambda})\,,$$
$$\mathrm{diag}\{\mathrm{diag}(\boldsymbol{SR}_{hh}\boldsymbol{S}^{\top}\boldsymbol{\Lambda})\}\overline{\boldsymbol{c}_d}+\boldsymbol{D}\overline{\boldsymbol{c}_d}=\mathrm{diag}(\boldsymbol{SR}_{hb}\boldsymbol{\Lambda})\,, \tag{S21}$$
$$\overline{\boldsymbol{c}_d}=\left[\mathrm{diag}\left\{\mathrm{diag}\left(\boldsymbol{SR}_{hh}\boldsymbol{S}^{\top}\boldsymbol{\Lambda}\right)\right\}+\boldsymbol{D}\right]^{-1}\mathrm{diag}(\boldsymbol{SR}_{hb}\boldsymbol{\Lambda})\,.$$

Here, we used the fact that the Hadamard product for a vector can be expressed as the product of a diagonal matrix and a vector. Finally, we derive Eq. (10) by substituting $\boldsymbol{\Phi}$ in Eq. (11) to Eq. (S21).

Supplementary Mathematical Derivations B. Estimating hyperparameters by empirical Bayes

In this section, we derive the hyperparameters λ and ρ by empirical Bayes. This is achieved by maximizing the free energy of Eq. (S1) with respect to each hyperparameter.

Hyperparameter $\boldsymbol{\lambda}$

We estimate the scaling parameter λ for the diagonal noise precision matrix $\boldsymbol{\Lambda} = \lambda \boldsymbol{I}$. To maximize the free energy of Eq. (S1) with respect to λ, we differentiate $\mathcal{F}$ by λ. Therefore, we first modify Eq. (S1) by summarizing terms not involved in the differentiation to ξ:

$$\mathcal{F}[\lambda] = \langle\langle \log P(\boldsymbol{H}, \boldsymbol{c}_d, \boldsymbol{B}) \rangle\rangle + \xi ,$$

$$= \langle\langle \log P(\boldsymbol{B} | \boldsymbol{H}, \boldsymbol{c}_d) \rangle\rangle + \xi , \qquad \text{(S22)}$$

$$= \frac{T}{2}\log|\boldsymbol{\Lambda}| - \frac{1}{2}\textstyle\sum_{t=1}^{T} \langle\langle \{\boldsymbol{b}(t) - \boldsymbol{CSh}(t)\}^{\top} \boldsymbol{\Lambda} \{\boldsymbol{b}(t) - \boldsymbol{CSh}(t)\} \rangle\rangle + \xi .$$

Each term on the right-hand side of Eq. (S22) is differentiated as follows:

$$\frac{\partial}{\partial\lambda}\left\{\frac{T}{2}\log|\boldsymbol{\Lambda}|\right\} = \frac{T}{2}\frac{\partial}{\partial\lambda}\log\lambda^{M} ,$$

$$= \frac{TM}{2\lambda}, \qquad \text{(S23)}$$

and

$$\frac{\partial}{\partial\lambda}\left\{-\frac{1}{2}\sum_{t=1}^{T} \langle\langle \{\boldsymbol{b}(t) - \boldsymbol{CSh}(t)\}^{\top} \boldsymbol{\Lambda} \{\boldsymbol{b}(t) - \boldsymbol{CSh}(t)\} \rangle\rangle \right\}$$

$$= -\frac{1}{2}\sum_{t=1}^{T} \langle\langle \{\boldsymbol{b}(t) - \boldsymbol{CSh}(t)\}^{\top} \{\boldsymbol{b}(t) - \boldsymbol{CSh}(t)\} \rangle\rangle , \qquad \text{(S24)}$$

$$= -\frac{1}{2}\sum_{t=1}^{T} \left\{ \boldsymbol{b}^{\top}(t)\boldsymbol{b}(t) - 2\boldsymbol{b}^{\top}(t)\overline{\boldsymbol{C}}\boldsymbol{S}\langle\boldsymbol{h}(t)\rangle + \mathrm{tr}\left[\boldsymbol{S}^{\top}\overline{\boldsymbol{C}^{2}}\boldsymbol{S}\langle\boldsymbol{h}(t)\boldsymbol{h}^{\top}(t)\rangle\right] \right\}.$$

By substituting Eqs. (S23) and (S24) to the differentiation of Eq. (S22), we obtain

$$\frac{\partial}{\partial\lambda}\mathcal{F}[\lambda] = \frac{TM}{2\lambda} - \frac{1}{2}\sum_{t=1}^{T} \left\{ \boldsymbol{b}^{\top}(t)\boldsymbol{b}(t) - 2\boldsymbol{b}^{\top}(t)\overline{\boldsymbol{C}}\boldsymbol{S}\langle\boldsymbol{h}(t)\rangle \right.$$

$$\left. + \,\mathrm{tr}\left[\boldsymbol{S}^{\top}\overline{\boldsymbol{C}^{2}}\boldsymbol{S}\langle\boldsymbol{h}(t)\boldsymbol{h}^{\top}(t)\rangle\right] \right\}. \qquad \text{(S25)}$$

Putting 0 on the left side, we obtain the estimated hyperparameter $\hat{\lambda}$:

$$\frac{TM}{2\hat{\lambda}} = \frac{1}{2}\sum_{t=1}^{T}\left\{\boldsymbol{b}^{\top}(t)\boldsymbol{b}(t) - 2\boldsymbol{b}^{\top}(t)\bar{\boldsymbol{C}}\boldsymbol{S}\langle\boldsymbol{h}(t)\rangle + \mathrm{tr}\left[\boldsymbol{S}^{\top}\overline{\boldsymbol{C}^{2}}\boldsymbol{S}\langle\boldsymbol{h}(t)\boldsymbol{h}^{\top}(t)\rangle\right]\right\},$$

$$\hat{\lambda}^{-1} = \frac{1}{TM}\sum_{t=1}^{T}\left\{\boldsymbol{b}^{\top}(t)\boldsymbol{b}(t) - 2\boldsymbol{b}^{\top}(t)\bar{\boldsymbol{C}}\boldsymbol{S}\langle\boldsymbol{h}(t)\rangle + \mathrm{tr}\left[\boldsymbol{S}^{\top}\overline{\boldsymbol{C}^{2}}\boldsymbol{S}\langle\boldsymbol{h}(t)\boldsymbol{h}^{\top}(t)\rangle\right]\right\},$$

$$= \frac{1}{TM}\sum_{t=1}^{T}\left\{\boldsymbol{b}^{\top}(t)\boldsymbol{b}(t) - 2\boldsymbol{b}^{\top}(t)\bar{\boldsymbol{C}}\boldsymbol{S}\bar{\boldsymbol{h}}(t) + \mathrm{tr}\left[\boldsymbol{S}^{\top}\overline{\boldsymbol{C}^{2}}\boldsymbol{S}\left(\boldsymbol{\Gamma}^{-1} + \bar{\boldsymbol{h}}(t)\bar{\boldsymbol{h}}^{\top}(t)\right)\right]\right\}, \tag{S26}$$

$$= \frac{1}{TM}\left\{\sum_{t=1}^{T}\boldsymbol{b}^{\top}(t)\boldsymbol{b}(t) - 2\sum_{t=1}^{T}\boldsymbol{b}^{\top}(t)\bar{\boldsymbol{C}}\boldsymbol{S}\bar{\boldsymbol{h}}(t) + \mathrm{tr}\left(\boldsymbol{S}^{\top}\overline{\boldsymbol{C}^{2}}\boldsymbol{S}\boldsymbol{R}_{hh}\right)\right\}.$$

Here, we used $\langle\boldsymbol{h}(t)\rangle = \bar{\boldsymbol{h}}(t)$, $\langle\boldsymbol{h}(t)\boldsymbol{h}^{\top}(t)\rangle = \boldsymbol{\Gamma}^{-1} + \bar{\boldsymbol{h}}(t)\bar{\boldsymbol{h}}^{\top}(t)$, and $\boldsymbol{R}_{hh} = T\boldsymbol{\Gamma}^{-1} + \sum\left(\bar{\boldsymbol{h}}(t)\bar{\boldsymbol{h}}^{\top}(t)\right)$.

Hyperparameter ρ

By adopting the same strategy used for λ, we estimate the scaling parameter ρ for the covariance matrix $\boldsymbol{D}^{-1} = \rho\boldsymbol{K}$ in Eq. (7).

First, we rewrite Eq. (S1) for ρ:

$$\begin{aligned}\mathcal{F}[\rho] &= \langle\langle\log P(\boldsymbol{c}_d)\rangle\rangle + \xi,\\ &= -M\log\rho - \log|\boldsymbol{K}| - \frac{1}{2\rho}\langle\boldsymbol{c}_d^{\top}\boldsymbol{K}^{-1}\boldsymbol{c}_d\rangle + \xi,\\ &= -M\log\rho - \frac{1}{2\rho}\mathrm{tr}\{\boldsymbol{K}^{-1}\langle\boldsymbol{c}_d\boldsymbol{c}_d^{\top}\rangle\} + \xi,\\ &= -M\log\rho - \frac{1}{2\rho}\mathrm{tr}\{\boldsymbol{K}^{-1}(\boldsymbol{\Phi}^{-1} + \overline{\boldsymbol{c}_d}\,\overline{\boldsymbol{c}_d}^{\top})\} + \xi.\end{aligned} \tag{S27}$$

Here, we used $\langle\boldsymbol{c}_d\boldsymbol{c}_d^{\top}\rangle = \boldsymbol{\Phi}^{-1} + \overline{\boldsymbol{c}_d}\,\overline{\boldsymbol{c}_d}^{\top}$. Then, Eq. (S27) is differentiated as

$$\frac{\partial}{\partial\rho}\mathcal{F}[\rho] = -M\rho^{-1} + \frac{1}{2}\rho^{-2}\mathrm{tr}\left\{\boldsymbol{K}^{-1}\left(\boldsymbol{\Phi}^{-1} + \overline{\boldsymbol{c}_d}\,\overline{\boldsymbol{c}_d}^{\top}\right)\right\}. \tag{S28}$$

Putting 0 on the left side, we obtain the estimated hyperparameter $\hat{\rho}$:

$$\hat{\rho} = \frac{1}{2M}\mathrm{tr}\left\{\boldsymbol{K}^{-1}\left(\boldsymbol{\Phi}^{-1} + \overline{\boldsymbol{c}_d}\,\overline{\boldsymbol{c}_d}^{\top}\right)\right\}. \tag{S29}$$

Supplementary Mathematical Derivations C. Computing the free energy

As explained in Supplementary Mathematical Derivations A, the variational Bayesian EM algorithm uses the free energy of Eq. (S1) as the cost function. To confirm convergence, monitoring the free energy is important. Moreover, it is one of the commonly used statistical criteria for model selection. Here, we derive the free energy for computing by rewriting Eq. (S1) as follows:

$$\begin{aligned}\mathcal{F} = \iint Q(\boldsymbol{H}|\boldsymbol{B})Q(\boldsymbol{c}_d|\boldsymbol{B}) \log P(\boldsymbol{H}, \boldsymbol{c}_d, \boldsymbol{B})\, d\boldsymbol{h} d\boldsymbol{c}_d \\ + \mathcal{H}[Q(\boldsymbol{H}|\boldsymbol{B})] + \mathcal{H}[Q(\boldsymbol{c}_d|\boldsymbol{B})]\,,\end{aligned} \tag{S30}$$

where $\mathcal{H}$ stands for an entropy $\mathcal{H}[P(x)] = -\int P(x) \log P(x) dx$. Each entropy term is calculated using the distribution at the end of every step of the EM algorithm:

$$\begin{aligned}\mathcal{H}[Q(\boldsymbol{H}|\boldsymbol{B})] &= -\frac{T}{2} \log|\boldsymbol{\Gamma}|\,, \\ \mathcal{H}[Q(\boldsymbol{c}_d|\boldsymbol{B})] &= -\frac{1}{2} \log|\boldsymbol{\Phi}|\,.\end{aligned} \tag{S31}$$

According to the definition of each distribution, the first term in Eq. (S30) is expanded as follows:

$$\begin{aligned}&\iint Q(\boldsymbol{H}|\boldsymbol{B})Q(\boldsymbol{c}_d|\boldsymbol{B}) \log P(\boldsymbol{H}, \boldsymbol{c}_d, \boldsymbol{B})\, d\boldsymbol{h} d\boldsymbol{c}_d \\ &= -\frac{1}{2}\sum_{t=1}^{T} [\langle\langle\{\boldsymbol{b}(t) - \boldsymbol{CSh}(t)\}^{\top} \boldsymbol{\Lambda} \{\boldsymbol{b}(t) - \boldsymbol{CSh}(t)\}\rangle\rangle \\ &\qquad - \langle \boldsymbol{h}^{\top}(t)\boldsymbol{h}(t)\rangle] - \frac{1}{2\rho}\langle \boldsymbol{c}_d^{\top}(t)\boldsymbol{c}_d(t)\rangle + \text{const.}, \\ &= -\frac{1}{2}\sum_{t=1}^{T} \{\boldsymbol{b}^{\top}(t)\boldsymbol{\Lambda}\boldsymbol{b}(t) - \bar{\boldsymbol{h}}^{\top}(t)\boldsymbol{\Gamma}\bar{\boldsymbol{h}}(t) + N\} \\ &\qquad - \frac{1}{2\rho}\mathrm{tr}\left(\boldsymbol{\Phi}^{-1} + \overline{\boldsymbol{c}_d}\,\overline{\boldsymbol{c}_d}^{\top}\right) + \text{const.}, \\ &\text{const.} = \frac{1}{2}\{T \log|\boldsymbol{\Lambda}| - M \log \rho - (MT + NT + M) \log 2\pi\}\,.\end{aligned} \tag{S32}$$

Here, the constant terms are not discarded to consider model comparisons using free energy. By substituting Eqs. (S31) and (S32) to Eq. (S30), we can compute the free energy:

$$
\begin{aligned}
\mathcal{F} = -\frac{1}{2}\Bigg[\sum_{t=1}^{T}\Big\{&-\boldsymbol{b}^{\top}(t)\boldsymbol{\Lambda}\boldsymbol{b}(t) + \overline{\boldsymbol{h}}^{\top}(t)\boldsymbol{\Gamma}\overline{\boldsymbol{h}}(t)\Big\} \\
&- \frac{1}{\rho}\operatorname{tr}\left(\boldsymbol{\Phi}^{-1} + \overline{\boldsymbol{c}_d}\,\overline{\boldsymbol{c}_d}^{\top}\right) \\
&+ T\log\frac{|\boldsymbol{\Lambda}|}{|\boldsymbol{\Gamma}|} + \log\left|\boldsymbol{\Phi}^{-\mathbf{1}}\right| - M\log\rho \\
&- (MT + NT + M)\log 2\pi - NT\Bigg].
\end{aligned}
\tag{S33}
$$

Supplementary Note 1: Mitigating mutual indeterminacy between H and C

Since the estimated values of $\boldsymbol{H}$ and $\boldsymbol{C}$ by VBCSSP are mutually adjustable, their absolute values are meaningless. To mitigate this issue, we introduce a correction based on the norm of the SSP estimate:

$$\boldsymbol{H}_{\mathrm{VBCSSP}} \leftarrow \alpha \boldsymbol{H}_{\mathrm{VBCSSP}},$$

$$\boldsymbol{c}_d \leftarrow \boldsymbol{c}_d/\alpha,$$

where $\alpha = \|\boldsymbol{H}_{\mathrm{SSP}}\|_F/\|\boldsymbol{H}_{\mathrm{VBCSSP}}\|_F$ is a correction factor, $\boldsymbol{c}_d$ is estimated channel gain vector by VBCSSP, $\boldsymbol{H}_{\mathrm{SSP}}$ and $\boldsymbol{H}_{\mathrm{VBSSP}}$ are estimated interference amplitudes by SSP and VBCSSP, $\|\ \ \|_F$ represents the Frobenius norm, and a left-facing arrow indicates replacing the left side with the right side.

To investigate the effect of this correction, we evaluated the mean of the estimated $\boldsymbol{c}_d$ using the static channel gain simulation data from Section 3.1 of the main text. Note that the mean of the diagonal components of $\boldsymbol{C}$ was set to 1 in the simulation. Fig. S 1 shows the results of VBCSSP with correction (VBCSSP with reg.) and without correction (VBCSSP w/o reg.). The CSSP estimate is included for comparison. Since CSSP is based on $\boldsymbol{H}_{\mathrm{SSP}}$ and has no iteration, there is no mutual indeterminacy, and thus the mean is always 1. VBCSSP without correction shows a significant decrease in the mean, which becomes more pronounced as L_{SSP} increases. On the other hand, when the correction was applied, the mean improved significantly and was comparable to the CSSP results up to $L_{\mathrm{SSP}} = 3$. However, when $L_{\mathrm{SSP}} \geq 4$, the mean decreased significantly. This becomes more pronounced as the gain error increases.

The above results suggest that the mutual indeterminacy problem between $\boldsymbol{H}$ and $\boldsymbol{C}$ in VBCSSP can be reasonably corrected if L_{SSP} is less than or equal to 3.

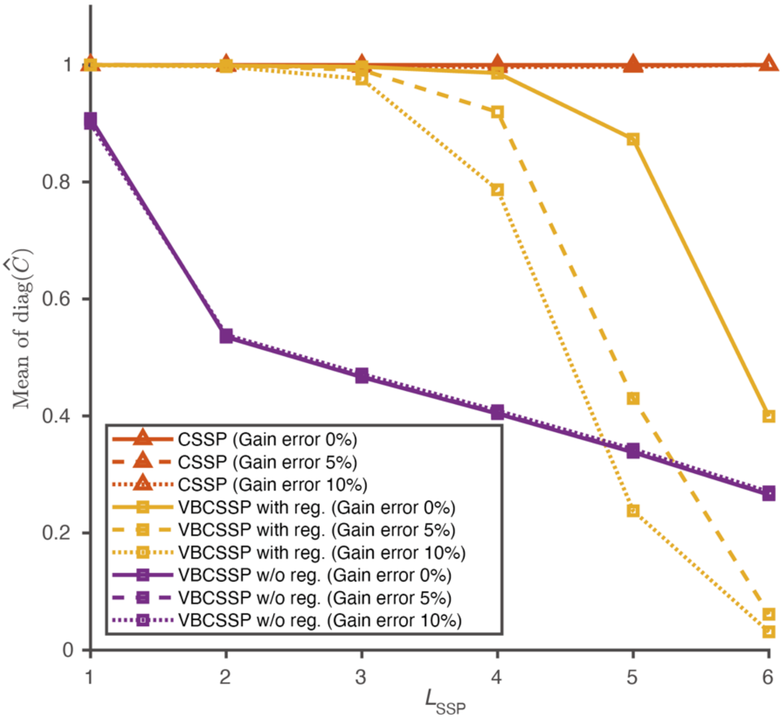

Fig. S 1 Investigating the effect of correction.

Mean of the diagonal components of estimated channel gain is plotted as a function of L_{SSP}. VBCSSP with correction (VBCSSP with reg.) and without correction (VBCSSP w/o reg.) are compared with CSSP. The results are visualized for gain errors of 0%, 5%, and 10%.

Supplementary Note 2: Analysis with real dataset of Rier et al. (2023)

Materials and methods

We evaluated subject identifiability based on connectome using a previously reported dataset (Rier et al., 2023). For this dataset's construction, ten subjects participated in a movie-watching task using 56 tri-axial OPM sensors (total 168 channels). A 10-minute run was repeated twice, and the reproducibility of connectome between runs was assessed. We computed the connectome and SF of the following bands: δ(2–4 Hz), θ(4–8 Hz), β(13–30 Hz), γ_1(30–40 Hz), γ_2(35–45 Hz), and γ_3(40–48 Hz). The connectome was computed using the amplitude envelope's functional connectivity (FC) (Brookes et al., 2011; O'Neill et al., 2015) after narrow-band beamforming (Hillebrand and Barnes, 2005) and cortical region-wise orthogonalization (Brookes et al., 2012).

In addition to SSP and VBCSSP, we also employed adaptive multipole models (AMMs) (Tierney et al., 2024) for comparison. Specifically, AMM was performed both as a spatial projector and via its temporal expansion to remove temporally correlated components. Aside from AMM, interference subspace was defined by the regular spherical harmonics function (Tierney et al., 2022); in the case of AMM, spheroidal harmonics was employed to model the neural and interference subspaces in place of the spherical harmonic function.

For each run, subject, and frequency band, we calculated the connectome matrix. To evaluate their similarity, we calculated the correlation coefficient of the connectome matrices between the two runs in the same subject (within-subject correlation coefficients) and between the two runs in different subjects (between-subject correlation coefficients). Then, we calculated the differences between the within- and between-subject correlation coefficients and averaged them across the subjects. This difference would be large if the test-retest reliability were high. Finally, we evaluated subject identifiability using connectome by counting the number of subjects showing a higher correlation coefficient for within-subject conditions than for between-subject conditions.

The analysis was performed using the scripts by Rier et al. in the following pipeline: filtering (bandpass 1–150 Hz and bandstop 50 Hz), manual channel and segmentation rejection, independent component analysis (ICA) (Makeig et al., 1996), HFC, bandpass filtering for each band, narrow-band beamforming, orthogonalization, and computing amplitude envelope FC. HFC in the pipeline was replaced by one of the following methods: SSP, VBCSSP, AMM, or AMM with temporal expansion (AMMt). The characteristics of each method are

summarized in the table below.

Table S 1 Summary of each method.

	SSP	CSSP	VBCSSP	AMM	AMMt
Gain estimation	No	Yes	Yes	No	No
Iterative optimization	No	No	Yes	No	No
Interference subspace	Spherical harmonics	Spherical harmonics	Spherical harmonics	Spheroidal harmonics	Spheroidal harmonics
Neural subspace	None	None	None	Spheroidal harmonics	Spheroidal harmonics
Temporal expansion	No	No	No	No	Yes

Results

First, we attempted to select the optimal L_{SSP} using the free energy criterion (Fig. S 2). The free energy was maximal for $L_{\mathrm{SSP}} = 1$ and decreased monotonically with increasing order, and this trend was consistent across both runs. Based on these findings, in the subsequent analysis, the interference subspace was modeled using first-order spherical or spheroidal harmonic functions, while for AMM and AMMt the neural subspace was modeled using ninth-order harmonics. In one subject (subject 10), VBCSSP rejected two channels, which were tangential axes of the same sensor.

Next, the methods were compared using SF (Fig. S 3). This was computed using Eq. (24) for each frequency and then averaged within the δ to γ_3 bands. SSP and VBCSSP yielded comparable SF, although VBCSSP exhibited a significantly higher SF than SSP in the δ band ($p < 0.05$). AMM and AMMt exhibited increased SF, particularly in the high-frequency bands.

Then, test-retest reliability was evaluated using the correlation coefficient of connectomes (Fig. S 4). We computed within- and between-subject correlation coefficients of the connectome between runs for each frequency band (a) and then computed the averaged difference between the two conditions (b). Each method showed a higher averaged correlation for within-subject conditions than between-subject conditions for all bands. In particular, apparent within-between differences were observed in the θ, α, and β bands. VBCSSP showed a slightly larger difference between averaged within- and between-subject

correlations than did SSP and AMM in the δ band. This result suggests that removing the low-frequency interference field by VBCSSP accentuated the differences between conditions. AMM and AMMt did not show noticeable improvements (Fig. S 3).

Finally, we assessed subject identifiability based on test-retest reliability (Fig. S 5). For VBCSSP, the scores were nearly identical to those obtained with SSP. AMM showed decreased scores in the δ, α, and γ_3 bands compared to SSP. AMMt showed improvement in the δ, γ_2, and γ_3 bands.

In summary, VBCSSP showed a slight improvement in SF compared to SSP but demonstrated equivalent performance in test-retest reliability of the connectome. On the other hand, while AMM remarkably improved SFs, it had a negative impact on subject identifiability based on test-retest reliability. However, by incorporating time expansion, AMMt improved subject identifiability as well.

We also confirmed the reproducibility of estimated channel gain across runs. Examples of the estimated channel gains for both runs are visualized in Fig. S 7. The correlation coefficient between runs was high (0.88 ± 0.03 [mean $\pm$ standard error of mean]), indicating the high robustness of VBCSSP.

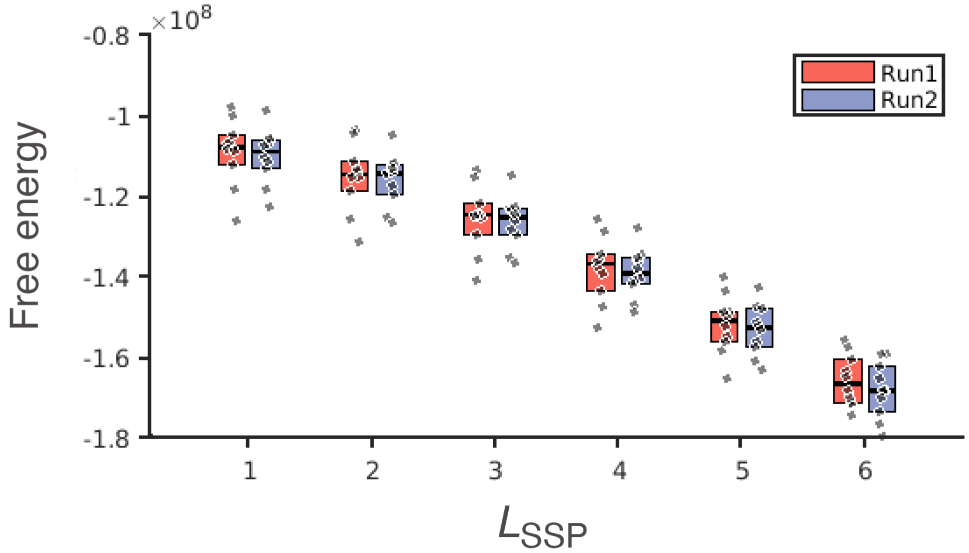

Fig. S 2 Free energy.

The horizontal axis is the order of spherical harmonics L_{SSP}, and the vertical axis is the free energy. Colored bars represent the results of the corresponding run.

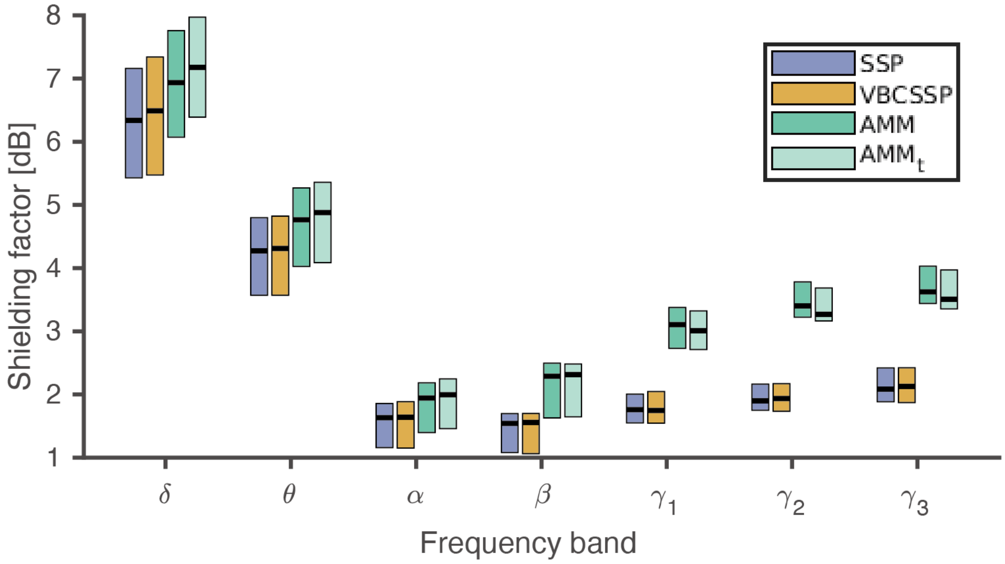

Fig. S 3 Shielding factor in each frequency band.

The horizontal axis is for each frequency band, and the vertical axis is the shielding factor SF. SF was computed by Eq. (24) and averaged for each band. Colored bars represent the results of the corresponding preprocessing method. Each dot represents the SF of a run (20 dots per band). The asterisk indicates a significant difference with $p<0.05$.

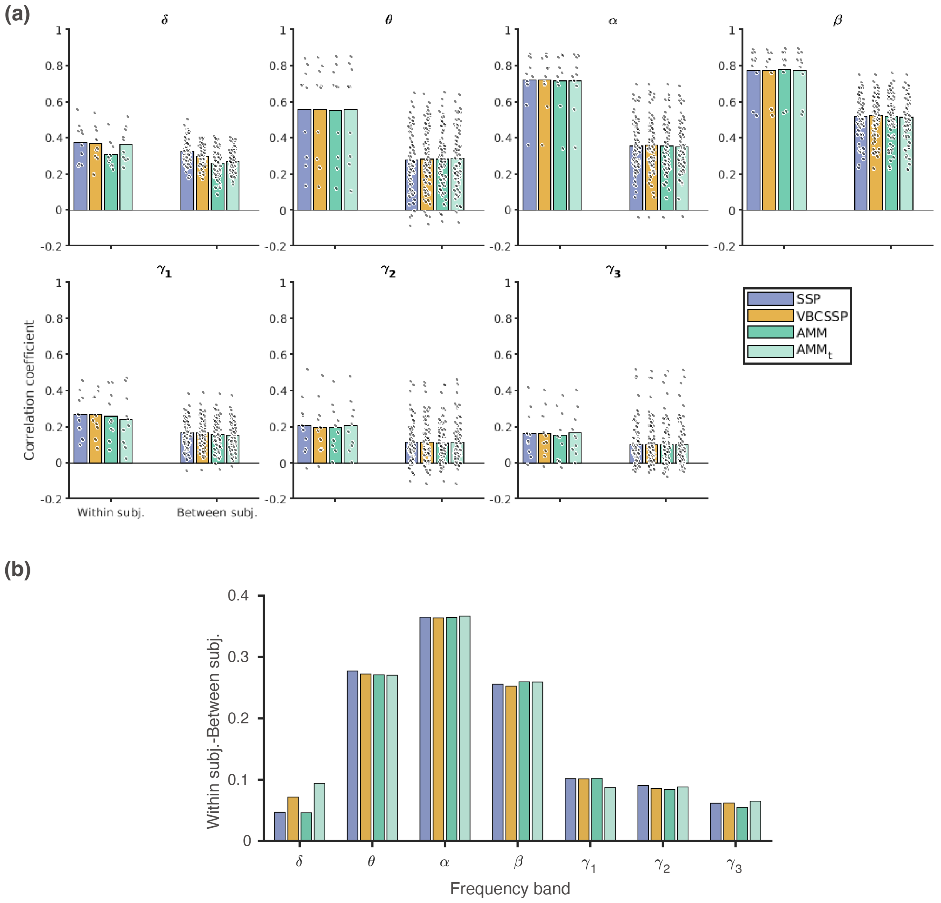

Fig. S 4 Test-retest reliability of connectome (a) and the difference between within- and between-subject correlations (b) for each frequency band.

(a): Within- and between-subject correlation coefficients of the connectome between runs are visualized for each frequency band. Each panel shows the results corresponding to a specific frequency band, where the vertical axis represents the correlation coefficient of the connectome between runs. Within- and between-subject correlations are summarized separately. (b): Difference between averaged within- and between-subject correlations. Colored bars represent the results of the corresponding preprocessing method.

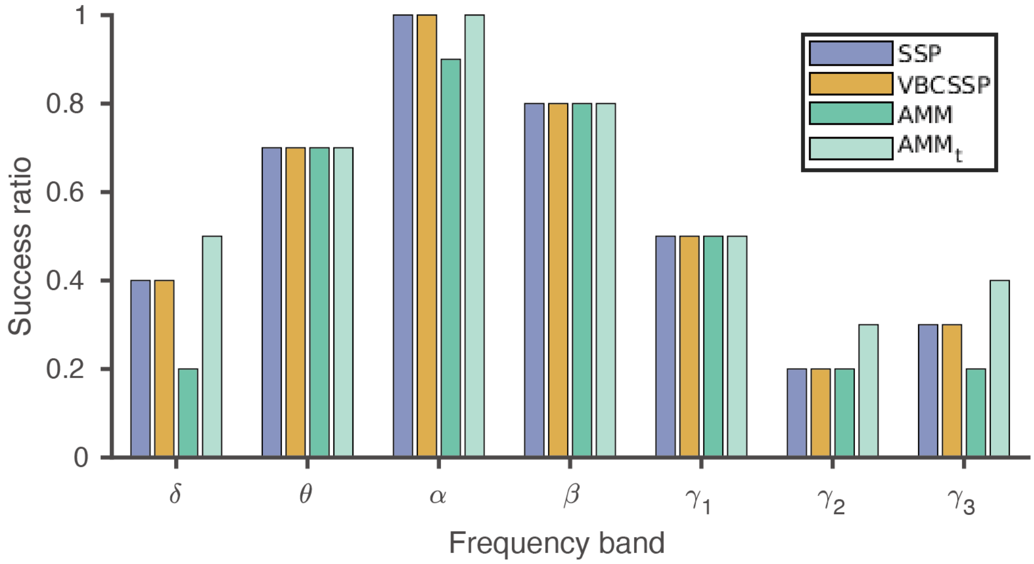

Fig. S 5 Subject identifiability using connectome in each frequency band.

The horizontal axis shows each frequency band, and the vertical axis is the number of subjects showing a higher correlation coefficient for within-subject conditions than between-subject conditions. Colored bars represent the results of the corresponding preprocessing method.

Supplementary Note 3: Topological maps of estimated channel gain for real datasets

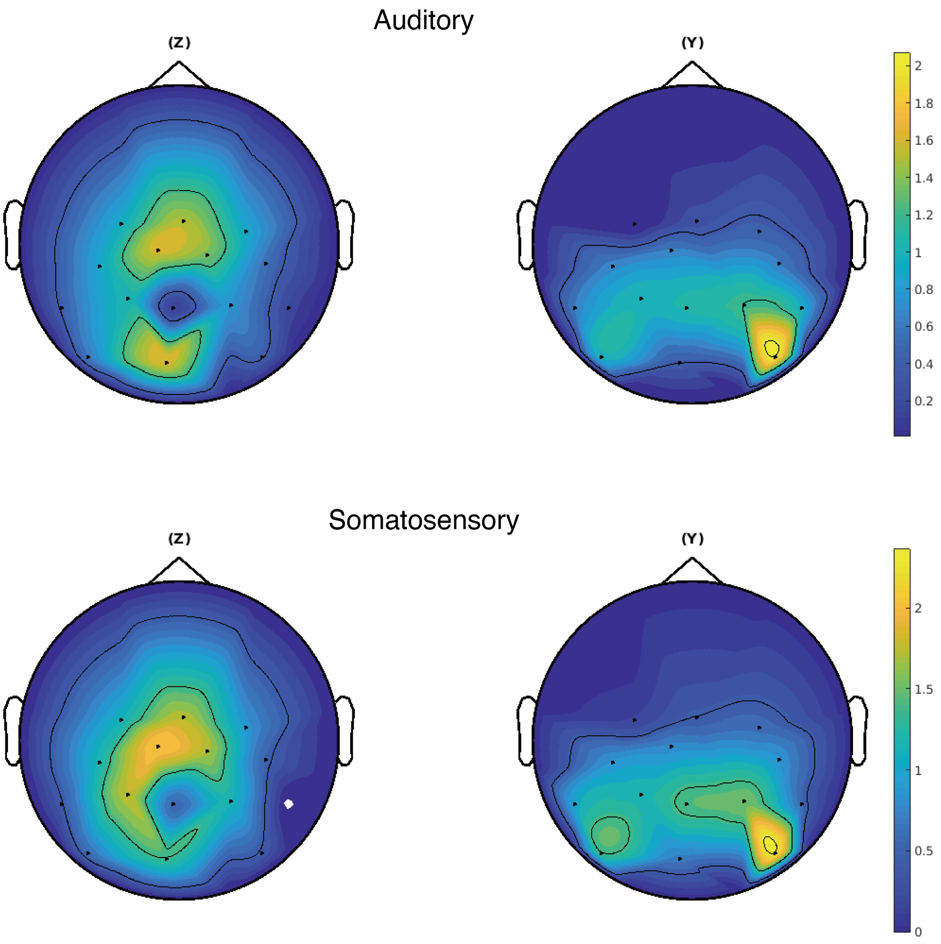

Fig. S 6 Topographic maps of estimated channel gain for the human experimental dataset described in main text.

The estimated channel gains for auditory (top) and somatosensory (bottom) tasks are visualized on each axis. Black dots indicate channel locations, and white dot indicates the channel rejected by VBCSSP.

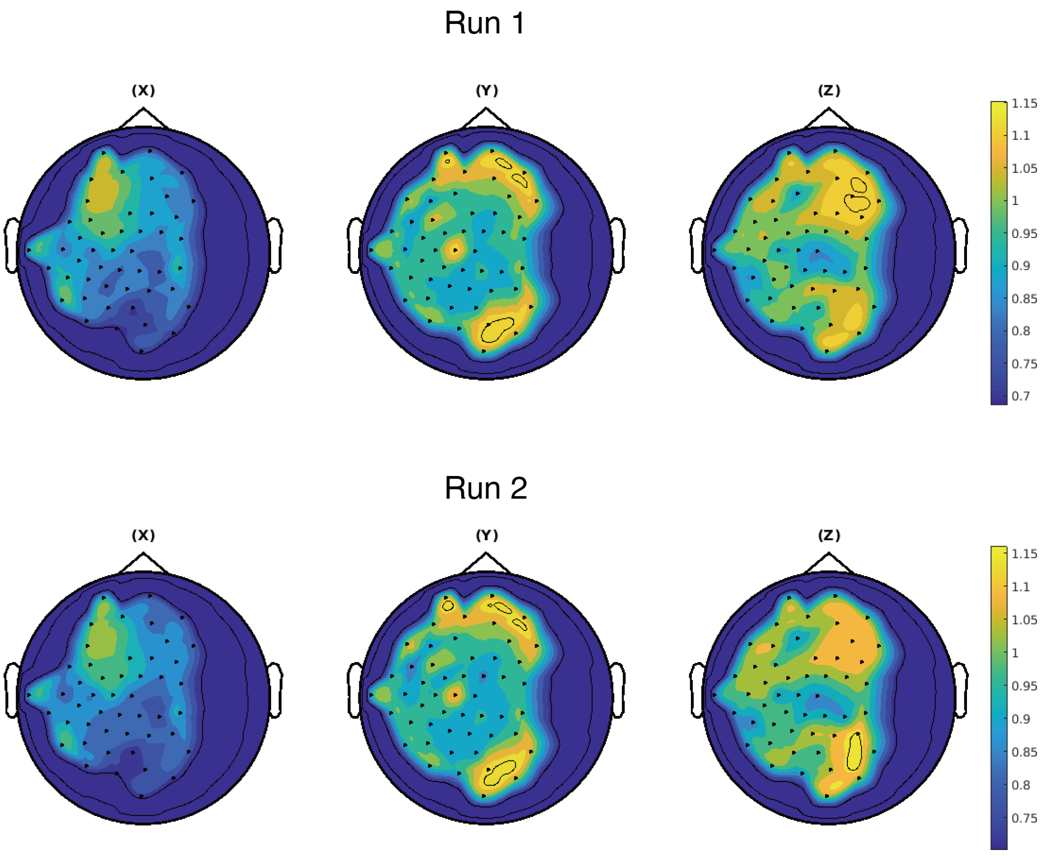

Fig. S 7 Topographic maps of estimated channel gain for the dataset in Supplementary Note 2.

The estimated channel gains for run 1 (top) and run 2 (bottom) are visualized on each axis. Black dots indicate channel locations.
